# Substrate-Induced Dimerization of Engineered Monomeric Variants of Triosephosphate Isomerase from *Trichomonas vaginalis*

**DOI:** 10.1371/journal.pone.0141747

**Published:** 2015-11-30

**Authors:** Samuel Lara-Gonzalez, Priscilla Estrella, Carmen Portillo, María E. Cruces, Pedro Jimenez-Sandoval, Juliana Fattori, Ana C. Migliorini-Figueira, Marisol Lopez-Hidalgo, Corina Diaz-Quezada, Margarita Lopez-Castillo, Carlos H. Trasviña-Arenas, Eugenia Sanchez-Sandoval, Armando Gómez-Puyou, Jaime Ortega-Lopez, Rossana Arroyo, Claudia G. Benítez-Cardoza, Luis G. Brieba

**Affiliations:** 1 IPICYT, División de Biología Molecular, Camino a la Presa San José 2055, CP 78216, San Luis Potosí, San Luis Potosí, México; 2 Laboratorio Nacional de Genómica para la Biodiversidad, Centro de Investigación y de Estudios Avanzados del IPN, Apartado Postal 629, CP 36500, Irapuato, Guanajuato, México; 3 Laboratorio de Investigación Bioquímica, Programa Institucional en Biomedicina Molecular ENMyH-IPN, Guillermo Massieu Helguera No. 239, La Escalera Ticoman, 07320, D.F, Mexico; 4 Laboratório Nacional de Biociências, Centro Nacional de Pesquisa em Energia e Materiais Campinas SP, Brazil; 5 Departamento de Bioquímica y Biología Estructural, Instituto de Fisiología Celular, Universidad Nacional Autónoma de México, Mexico City, México; 6 Departamento de Biotecnología y Bioingeniería, Centro de Investigación y de Estudios Avanzados del IPN, Col. San Pedro Zacatenco, Av. IPN, 2508, C.P. 07360, D.F., México; 7 Departamento de Infectómica y Patogénesis Molecular, Centro de Investigación y de Estudios Avanzados del IPN, Col. San Pedro Zacatenco, Av. IPN, 2508, C.P. 07360, D.F., México; University of Minnesota, UNITED STATES

## Abstract

The dimeric nature of triosephosphate isomerases (TIMs) is maintained by an extensive surface area interface of more than 1600 Å^2^. TIMs from *Trichomonas vaginalis* (TvTIM) are held in their dimeric state by two mechanisms: a ball and socket interaction of residue 45 of one subunit that fits into the hydrophobic pocket of the complementary subunit and by swapping of loop 3 between subunits. TvTIMs differ from other TIMs in their unfolding energetics. In TvTIMs the energy necessary to unfold a monomer is greater than the energy necessary to dissociate the dimer. Herein we found that the character of residue I45 controls the dimer-monomer equilibrium in TvTIMs. Unfolding experiments employing monomeric and dimeric mutants led us to conclude that dimeric TvTIMs unfold following a four state model denaturation process whereas monomeric TvTIMs follow a three state model. In contrast to other monomeric TIMs, monomeric variants of TvTIM1 are stable and unexpectedly one of them (I45A) is only 29-fold less active than wild-type TvTIM1. The high enzymatic activity of monomeric TvTIMs contrast with the marginal catalytic activity of diverse monomeric TIMs variants. The stability of the monomeric variants of TvTIM1 and the use of cross-linking and analytical ultracentrifugation experiments permit us to understand the differences between the catalytic activities of TvTIMs and other marginally active monomeric TIMs. As TvTIMs do not unfold upon dimer dissociation, herein we found that the high enzymatic activity of monomeric TvTIM variants is explained by the formation of catalytic dimeric competent species assisted by substrate binding.

## Introduction

Triosephosphate isomerase (TIM) is a (β/α)_8_ barrel enzyme that catalyzes the reversible conversion between glyceraldehyde-3-phosphate (G3P) and dihydroxyacetone phosphate (DHAP) near diffusion limit [1]. Proteins with a (β/α)_8_ fold are monomeric or oligomeric, but several reports conclude that TIM is an obligate dimer [2–5]. Its dimer interface consists of loop3 interdigitation between subunits and interactions between a set of conserved hydrophobic residues located in αhelices 2 and 3 [6–11]. Altered TIM dimerization is associated with human diseases [12, 13] and the notion that TIMs are catalytically inefficient as a monomer is key to develop drugs that disrupt their dimer interface [14–17]. Homodimers are prevalent in proteomes indicating that dimerization is a mechanism that minimizes the destabilizing effect of mutations [18–20]. *Trichomonas vaginalis* contains two fully functional TIMs that only differ in 4 out of 254 amino acids [21]. Although the high sequence identity between both TvTIMs, TvTIM1 requires 16.6 kJ mol^-1^ more energy for dimer dissociation and the only amino acid at the dimer interface that differs between both TIMs corresponds to I45 in TvTIM1 and V45 in TvTIM2 [21, 22]. Crystal structures of TvTIMs indicate that the protruding methyl of I45 in TvTIM1 fits into a hydrophobic pocket of the neighbor monomer, whereas V45 in TvTIM2 creates a cavity at the dimer interface. Cavities have a deleterious effect in protein stability and we rationalized that altering the van der Waals radius of I45 in TvTIM1 could create a cavity in the interface that may alter its dimeric nature [22].

## Material and Methods

### Amino acid sequence alignment and WEB logo

A multiple sequence alignment (using the MUSCLE algorithm [23]) of 433 amino acid sequences of TIMs covering Archaea, Eukarya and Bacteria in MEGA5 software[24] was used to generate the logo sequence using the web platform WebLogo (http://weblogo.berkeley.edu/) version 2.8.2 [25].

### Heterologous expression and purification

TvTIMs were purified as previously reported and were dialyzed against 20 mM Tris-HCl pH 7.4, 100 mM NaCl (Tris Buffer) or 100 mM triethanolamine pH 7.4, 100 mM NaCl (TEA buffer) and stored at 4°C [22]. Ball and socket mutants were constructed by Quick Change method as previously described [22].

### Kinetics parameters

The catalytic constants for the reverse reaction were calculated accordingly to the method of Plaut and Knowles [26]. Briefly TIM activity was assayed at 25°C using a coupled reaction in which D-glyceraldehyde 3-phosphate (DGAP) was used as a substrate. The product formed (dihydroxyacetone phosphate) was then reduced by α-glycerophosphate dehydrogenase (GDH) while NADH oxidation was detected by absorbance changes at 340 nm. The experimental procedure consisted in 1.0 mL reactions containing 100 mM triethanolamine buffer (pH 7.4), 10 mM EDTA, 0.20 mM NADH, 1.0 mM DGAP, and 0.01 mg of α-glycerophosphate dehydrogenase and DGAP concentration varied from 0.05 to 3.0 mM. The reactions started by adding TvTIMs. Kinetic parameters were calculated from the initial velocities at each substrate concentration. Wild-type and dimeric TvTIM were present at a final concentration of 5 ng/ml (~ 0.18 nM for the monomer). The concentration used for monomeric variants was increased because they exhibited lower activity. I45A, I45G, I45F and I45Y were present at 2, 5, 10 and 12 nM respectively.

### *In vivo* complementation studies

A ΔTIM strain was grown on minimal media agar plates lacking six carbon sugars as previously described [27]. These plates were supplemented with M63 salts and 0.2% w/v glycerol, 1 mg L^-1^ thiamine, 80 mg L^-1^, histidine and 50 mg L^-1^ uracil. Plates contained ampicillin for plasmid selection and kanamycin for strain selection. Cells were grown for four days at 37°C. Experiments in liquid media were done as follows: One single colony from every mutant grown in rich media was picked and inoculated in a LB media at 37°C overnight inoculums were centrifuged and pellets were washed with minimal media several times. Cultures were adjusted to 0.03 of OD_600_ in 96-well plates with triplicates for every strain. Plates were incubated at 37°C with shaking and OD_600_ was monitored during 40 hrs every hour in a TECAN reader spectrophotometer.

### Spectroscopic measurements

Fluorescence spectra were obtained using a LS-55 Spectrofluorometer (Perkin-Elmer), equipped with a water-jacketed cell holder for temperature control at 25°C. Fluorescence emission scans were recorded from 320 to 400 nm using an excitation wavelength of 280 nm (2.5 nm bandpass) with a 1 cm path-length cell. Samples were complemented with 8-Anilino-1-naphthalenesulfonic acid (ANS) at 1:50 molar ratio. ANS fluorescence was measured using an excitation and emission wavelengths of 360 and 460 nm respectively. Far-UV CD spectra were measured using a JASCO J-815 spectropolarimeter (Jasco Inc., Easton, MD) equipped with a PFD-425S Peltier-type cell holder for temperature control and magnetic stirring. Scans were taken between 200 to 250 nm, at a scan rate of 10 nm min^-1^ using a 1.0 cm path-length cuvette. Ellipticities are reported as mean residue ellipticity (θ]_MRW_).

### GdnHCl induced unfolding/refolding of triosephosphate isomerase mutants

Chemical denaturation experiments were performed using GdnHCl as denaturant and the methods described in detail for wild type TvTIMs [21]. The fluorescence spectral centre of mass (SCM) was calculated from the fluorescence intensity data (I_λ_), obtained at different wavelengths (λ) from 320 to 400 nm, using the equation [25]:

$$\text{SCM}=\Sigma \;(\lambda \;{\text{x I}}_{\lambda })\;/\;\Sigma {\text{ I}}_{\lambda }$$

### Data analysis

All data analysis was performed using the non-linear, least-squares fitting program Origin, version 8.0 (GraphPad Software).

#### Three-state monomer denaturation models

The chemical denaturation transitions of the monomeric constructs were fit to a three -state denaturation model:

$$N\overset{K_{D,1}}{\Leftrightarrow }I\overset{K_{D,2}}{\Leftrightarrow }D$$

where *N*, *I*, and *D* are native, intermediate, and unfolded protein, respectively. *K*_*D*,*1*_ and *K*_*D*,*2*_ are the equilibrium constants for each folding step, respectively. If the species present at each denaturant concentration are expressed as fraction (*f*), then the conservation of mass can be expressed as follows,

$$f_{N}+f_{I}+f_{D}=1$$ (1)

If the unfolding reaction is in equilibrium *f*_*N*_, *f*_*I*_ y *f*_*D*_ are related to *K*_*D*,*1*_ and *K*_*D*,*2*_ and in consequence with the free energy changes corresponding to the first reaction step, *ΔG*_*NI*_ and with the free energy change of the global reaction *ΔG*_*ND*_ as:

$$f_{N}=\frac{1}{\left(1+K_{NI}+K_{ND}\right)}=\frac{1}{\left[1+\left(\frac{-\Delta G_{NI}}{RT}\right)+\left(\frac{-\Delta G_{ND}}{RT}\right)\right]}$$ (2)

fI=KNI(1+KNI+KND)=(−ΔGNIRT)[1+exp(−ΔGNIRT)+(−ΔGNDRT)] (3)

$$f_{D}=\frac{K_{ND}}{\left(1+K_{NI}+K_{ND}\right)}=\frac{\left(\frac{-\Delta G_{ND}}{RT}\right)}{\left[1+\text{exp}\left(\frac{-\Delta G_{NI}}{RT}\right)+\left(\frac{-\Delta G_{ND}}{RT}\right)\right]}$$ (4)

It is assumed that free energy changes show a linear dependence on the denaturant concentration according to:

$$\Delta G_{NI}=\Delta G_{NI}^{H_{2}O}-m_{N}{}_{I}\left[D\right]$$ (5)

$$\Delta G_{ND}=\Delta G_{ND}^{H_{2}O}-m_{ND}\left[D\right]$$ (6)

where $\Delta G_{NI}^{H_{2}O}$ and $\Delta G_{ND}^{H_{2}O}$ are the free energy change in the absence of denaturant, and *m*_*NI*_ and *m*_*ND*_ represent constants of proportionality relating to the solvent exposure difference between native and denatured states. The measured signal (*y*) depend on the species composition at each denaturant concentration according to the following equation,

$$y=y_{N}f_{N}+y_{I}f_{I}+y_{D}f_{D}$$ (7)

where *y*_*N*_, *y*_*I*_, and *y*_*D*_ are the specific signal of native, intermediate, and unfolded protein, respectively. The fitting equation was obtained by combining Eqs 2, 3 and 4 with Eqs 5 and 6 and then by substituting into Eq 7:

$$y=\frac{y_{N}+y_{I}e\left[\frac{-\Delta G_{NI}^{H_{2}O}-m_{NI}[d]}{RT}\right]+y_{U}e\left[\frac{-\Delta G_{NU}^{H_{2}O}-m_{ND}[d]}{RT}\right]}{1+e\left[\frac{-\Delta G_{NI}^{H_{2}O}-m_{NI}[d]}{RT}\right]+e\left[\frac{-\Delta G_{NU}^{H_{2}O}-m_{ND}[d]}{RT}\right]}$$ (8)

#### Four-state dimer denaturation models

The GdnHCl-induced denaturation transitions of dimeric TvTIM constructs were globally fit over all concentrations of protein to a four-state dimer denaturation model involving two monomeric intermediates according to the model

$$N_{2}\overset{K_{D,1}}{\Leftrightarrow }2M\overset{K_{D,2}}{\Leftrightarrow }2I\overset{K_{D,3}}{\Leftrightarrow }2D$$

In this model, the protein is assumed to be in either the native homodimeric state (*N*_*2*_), two monomeric states depicted as *M* or *I*, or in an unfolded monomeric state (*D*), and *K*_D,1_, *K*_D,2_, and *K*_D,3_ are the equilibrium constants for the three steps, respectively.

We consider the total molar concentration of the polypeptide chains as *P*_t_, as

$$P_{t}=2[N_{2}]+[M]+[I]+[D]$$ (9)

then the mole fraction of each species can be defined as

$$f_{N_{2}}=\frac{2[N_{2}]}{P_{t}}$$ (10)

$$f_{M}=\frac{\left[M\right]}{P_{t}}$$ (11)

$$f_{I}{}_{}=\frac{\left[I\right]}{P_{t}}$$ (12)

$$f_{D}=\frac{\left[D\right]}{P_{t}}$$ (13)

The sum of all fractions, as in the previous case is equal to unity:

$$f_{N_{2}}+f_{M}+f_{I}+f_{D}=1$$ (14)

The equilibrium constants *K*_D,1_, *K*_D,2_, and *K*_D,3_ are related to the mole of fraction of each species and to *P*_*t*_ as

$$K_{D,1}=\frac{2P_{t}f_{M}^{2}}{f_{N_{2}}}$$ (15)

$$K_{D,2}=\frac{f_{M}}{f_{I}}$$ (16)

$$K_{D,3}{}_{}=\frac{f_{D}}{f_{I}}$$ (17)

$f_{N_{2}},$ *f*_*M*_ and *f*_*I*_ can be defined only in terms of *f*_*D*_, *K*_*D*,*1*_, *K*_*D*,*2*_, *K*_*D*,*3*_ and *Pt*. *f*_*D*_ can be expressed as a function of *K*_*D*,*1*_, *K*_*D*,*2*_, *K*_*D*,*3*_ and *P*_*t*_

$$f_{N}{}_{{}_{2}}=\frac{2P_{t}K_{D,2}^{2}}{K_{D,1}K_{D,3}^{2}}f_{D}^{2}$$ (18)

$$f_{M}=\frac{K_{D,2}}{K_{D,3}}f_{D}$$ (19)

$$f_{I}=\frac{f_{D}}{K_{D.3}}$$ (20)

We assume the free energy change for each step in the reaction to be linearly dependent on denaturant concentration as described earlier

$$-\Delta G_{D,1}=\Delta G_{D,1}^{H_{2}O}-m_{1}\left[denaturant\right]$$ (21)

$$-\Delta G_{D,2}=\Delta G_{D,2}^{H_{2}O}-m_{2}\left[denaturant\right]$$ (22)

$$-\Delta G_{D,3}=\Delta G_{D,3}^{H_{2}O}-m_{3}\left[denaturant\right]$$ (23)

where $\Delta G_{D,1}^{H_{2}O}$, $\Delta G_{D,2}^{H_{2}O}$ and $\Delta G_{D,3}^{H_{2}O}$ are the free energy changes in the absence of denaturant corresponding to *K*_*D*,*1*_, *K*_*D*,*2*_, *K*_*D*,*3*_, respectively, and *m*_1_, *m*_2_, and *m*_3_ are the cooperativity indices associated with each step. The amplitude of the spectroscopic signal determined at each denaturant concentration is assumed to be a linear combination of the fractional contribution from each species:

$$y=y_{N_{2}}f_{N_{2}}+y_{I_{2}}f_{I_{2}}+y_{I}f_{I}+y_{D}f_{D}$$ (24)

where $y_{N_{2}}$,$y_{I_{2}}$, *y*_I_, and *y*_U_ are the amplitudes of the signals for the respective species.

$$f_{D}=\frac{-\left(\frac{K_{D,2}}{K_{D,3}}+\frac{1}{K_{D,}{}_{3}}+1\right)\pm \sqrt{{\left(\frac{K_{D,2}}{K_{D,3}}+\frac{1}{K_{D,}{}_{3}}+1\right)}^{2}+8P_{t}\frac{K_{D,2}^{2}}{K_{D,1}K_{D,3}^{2}}}}{4P_{t}\left(\frac{K_{D,2}^{2}}{K_{D,1}K_{D,3}^{2}}\right)}$$ (25)

The fitting equation is then:

$$f_{D}=\frac{-\left(\frac{\left({\text{exp}}^{-\left(\frac{\Delta G_{D,2}^{H_{2}O}-m2\left[denaturant\right]}{RT}\right)}\right)}{{\left({\text{exp}}^{-\left(\frac{\Delta G_{D,3}^{H_{2}O}-m_{3}\left[denaturant\right]}{RT}\right)}\right)}_{}}+\frac{1}{\left({\text{exp}}^{-\left(\frac{\Delta G_{D,3}^{H_{2}O}-m_{3}\left[denaturant\right]}{RT}\right)}\right)}+1\right)\pm \sqrt{{\left(\frac{\left({\text{exp}}^{-\left(\frac{\Delta G_{D,2}^{H_{2}O}-m2\left[denaturant\right]}{RT}\right)}\right)}{\left({\text{exp}}^{-\left(\frac{\Delta G_{D,3}^{H_{2}O}-m_{3}\left[denaturant\right]}{RT}\right)}\right)}+\frac{1}{\left({\text{exp}}^{-\left(\frac{\Delta G_{D,3}^{H_{2}O}-m_{3}\left[denaturant\right]}{RT}\right)}\right)}+1\right)}^{2}+8P_{t}\frac{{\left({\text{exp}}^{-\left(\frac{\Delta G_{D,2}^{H_{2}O}-m2\left[denaturant\right]}{RT}\right)}\right)}_{}^{2}}{\left({\text{exp}}^{-\left(\frac{\Delta G_{D,1}^{H_{2}O}-m_{1}\left[denaturant\right]}{RT}\right)}\right){\left({\text{exp}}^{-\left(\frac{\Delta G_{D,3}^{H_{2}O}-m_{3}\left[denaturant\right]}{RT}\right)}\right)}_{}^{2}}}}{4P_{t}\left(\frac{{\left({\text{exp}}^{-\left(\frac{\Delta G_{D,2}^{H_{2}O}-m2\left[denaturant\right]}{RT}\right)}\right)}_{}^{2}}{\left({\text{exp}}^{-\left(\frac{\Delta G_{D,1}^{H_{2}O}-m_{1}\left[denaturant\right]}{RT}\right)}\right){\left({\text{exp}}^{-\left(\frac{\Delta G_{D,3}^{H_{2}O}-m_{3}\left[denaturant\right]}{RT}\right)}\right)}_{}^{2}}\right)}$$ (26)

### Crystallization, data collection and structure determination

Crystallization experiments were carried out as previously described [22]. A summary of data collection statistics and refinement is given in S1 Table. The molecular replacement search model consisted of the crystal structure of wild-type TvTIM1 lacking residues 30 to 90 to avoid model bias. Initial maps were used for rounds manual building and refinement.

### Partial Proteolysis

Partial Proteolysis was carried out at 30°C. Wild-type TvTIM1 and point mutants were diluted to a concentration of 1 mg/ml in 100 mM triethanolamine (pH 7.4) and incubated with trypsin at 1:500 molar ratio. Aliquots were taken at the indicated time points and quenched by the addition of 5 mM phenylmethylsulfonyl fluoride followed by SDS-loading buffer. Proteolysis was monitored by SDS-PAGE.

### Analytical ultracentrifugation in the presence of PGH

Protein concentration in samples was determined by absorbance in 280 nm using a spectrophotometer V-530/JASCO. AUC experiments were performed on an XL-A analytical ultracentrifuge (Beckman, Fullerton, CA) with an An-50 Ti rotor. The sedimentation velocity (SV) experiments were performed in a double-sector epon charcoal-filled centerpiece at 20°C with a rotor speed of 129,024 rcf. Sample (420 μL) and reference/buffer (440 μL) solutions with or without different concentrations of substrate analogs were loaded into the centerpiece. Absorbance at 280 nm was chosen to detect the protein, which was monitored in a continuous mode with a step size of 0.003 cm. Proteins were present at 1 mg/mL (35–40 μM, Absorbance _280nm_ ~0.8) free or with substrate analogs in three different concentrations (20, 250 and 600 μM). Around 120 scans at different time intervals were acquired and then fitted to a continuous c(s) distribution model using the SEDFIT program [28]. The partial specific volume of protein, the solvent density, and the viscosity were calculated by SEDNTERP [29].

### Cross-linking Reactions

Chemical cross-linking was performed using the homobifunctional cross-linking agent 1,8-bis-maleimidodiethyleneglycol (BM(PEG)2, Thermo scientific). The stock solution was prepared at a final concentration of 20 mM in DMSO. The solutions of purified proteins were complemented with the crosslinker to a final concentration of 0.2 mM (two-fold molar excess for 0.1mM protein). The mixture was incubated for 1 hour at room temperature. Afterwards, the un-reacted reagent was removed by gel filtration using a 24 ml Superdex 75 10/300 GL equilibrated in a buffer 100 mM triethanolamine (pH 7.4), 100 mM NaCl, 10 mM EDTA, 1 mM DTT.

## Results

**Fig 1:**
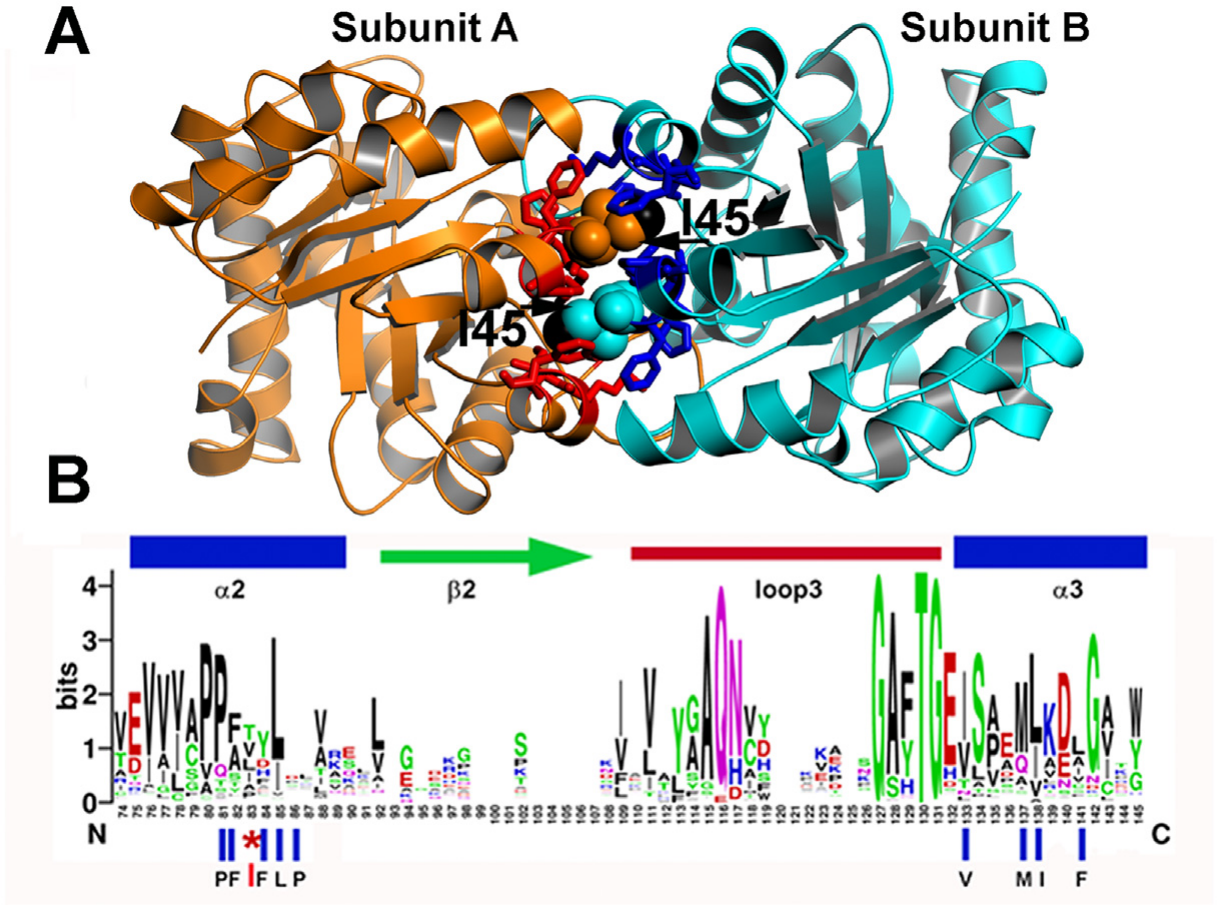
Ball and socket interaction between monomers at TvTIM1. **(A)** Crystal structure of TvTIM1 showing the ball and socket interplay. Hydrophobic TvTIM1 residues (ball-stick representation) form a socket that interacts with residue Ile45 of the neighboring subunit (space-filling representation). The methyl group of I45 is colored in black. **(B)** Sequence logo showing the structural alignment of the ball-socket amino acids at the ball and socket interplay Residue I45 or V45 functions as the ball and a hydrophobic cavity formed by α-helices 2 and 3 that assemble as the socket.

Residue I45 of TvTIM1 functions as a ball that fits into a hydrophobic cavity composed of residues P43, F44, F46, L47, P48, V77, M81, I82, and F85 (Fig 1A).

Among 420 amino acid sequences of TIMs, residues T, L, I, V, A and P account for 40% of the amino acids at position 45 [22]. Of the nine amino acids that comprise the socket only P43 and L47 are conserved (Fig 1B and S1 Fig).

Here we study whether residues with altered van der Waals radius (G, A, V, L, and W) would influence the dimer-monomer fate of TvTIM1. All mutants, with the exception of I45W were soluble. I45W was present in the insoluble fraction during its purification assays using all 30 lysis buffers of a sparse matrix solubility screen [30] (data not shown).

### Single point mutations of residue 45 results in monomeric enzymes

**Fig 2:**
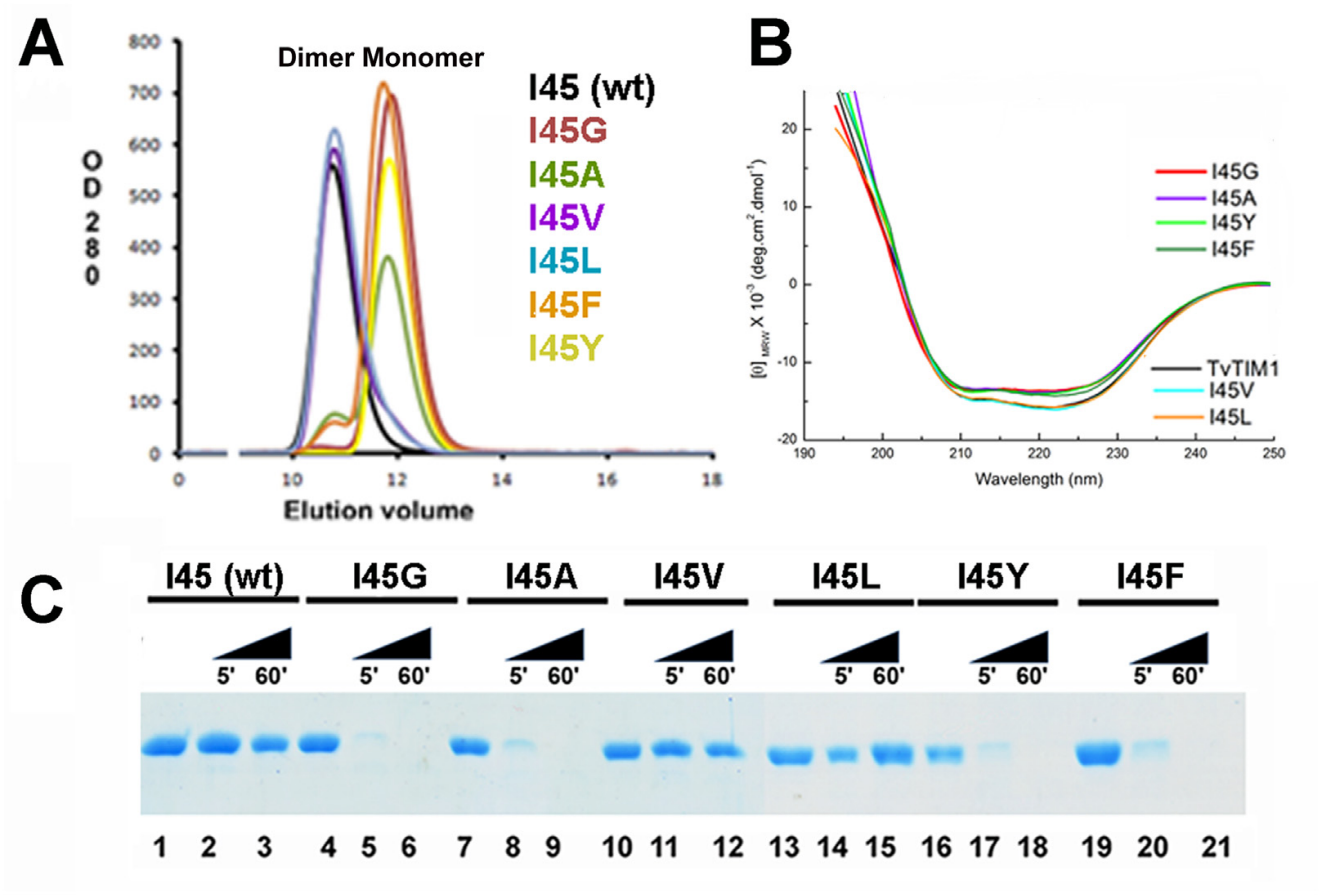
The identity of residue 45 determines the dimer-monomer equilibrium of TvTIM1. **(A)** Gel filtration elution profiles of wild-type and residue 45 point mutants. Mutants I45G, I45V, I45F and I45Y present as a monomer, whereas I45V and I45L as dimer. Mutant I45G and I45A present a small peak (6 and 3% of the total protein respectively) at the retention time of the dimer indicating that both mutants at a concentration of 260 μM exist in dimer-monomer equilibrium. **(B)** CD spectra of wild type and I45 mutants. The spectra of I45L and I45V superimpose with the wild-type spectra, whereas monomeric constructs present a decrease on ellipticity. **(C)** Partial proteolysis of TvTIM1 mutants. SDS-PAGE showing the digestion patterns of digested proteins after a partial proteolysis experiment (5 and 60 minutes). At 5 minutes almost the totality of the monomeric proteins I45G, I45V, I45F and I45Y are digested by trypsin, and after 60 minutes the digestion is complete. In contrast a strong protein band is observed for wild-type and of I45L and I45V mutants indicating that dimeric enzymes present an increased resistance to proteolysis.

We measured the oligomeric state of the ball-socket mutants using gel filtration at a concentration of 7 mg/ml (~260 μM) in Tris pH 7.4, 100 mM NaCl (Fig 2A).

Wild-type, I45V, and I45L mutants eluted in a single peak at an elution volume of 10.7 ml that corresponds to the retention volume of ~54 kDa. This contrasts to the elution profile of I45G, I45A, I45F and I45Y mutants. Mutants I45F and I45Y eluted in a single peak at 11.8 ml that corresponds to the molecular mass of TvTIM monomers (~27 kDa). I45A and I45F mutants were present in a dimer-monomer equilibrium showing two peaks, one at an elution volume that corresponds to the molecular mass of a monomer and other that corresponds to the molecular mass of a dimer. The dimer of I45A and I45F corresponds to 13% and 5.3% of the total injected protein respectively. I45F and I45A run at 26 μM eluted in a single peak corresponding the elution profiles of monomers (data not shown).

### Mutations in the ball present altered CD spectra and are susceptible to proteolysis

We were curious to assess if monomeric TvTIMs mutants may present structural deviations. The CD spectra of dimeric mutants I45L and I45V superimpose with the wild-type TvTIM1 spectra, whereas monomeric constructs show a small decrease on ellipticity centered at 222 nm (Fig 2B). The possible structural or oligomeric differences between wild-type and TvTIM mutants were also assessed by partial proteolysis. We observed that dimeric TvTIMs are partially resistant to trypsin after 1 hour of incubation (Fig 2C, lanes 3, 12, 15) and that this resistance extends for at least for 6 hours (data not show), whereas monomeric TvTIMs are susceptible and suffer complete proteolysis at 60 minutes of incubation (Fig 2C, lanes 5, 8, 17, 20).

### Monomeric ball mutants are active enzyme

To understand the significance of the altered oligomeric state of TvTIM mutants we measured their enzymatic activity employing glyceraldehyde phosphate as substrate. Wild-type and dimeric TvTIMs were present at 0.2 nM whereas I45A, I45G, I45F and I45Y were present at 2, 5, 10 and 12 nM respectively. The catalytic efficiency of dimeric mutants is similar to wild-type TvTIM1, whereas the catalytic efficiency of the monomeric variants varies from 7.3x10^2^ to 1.19x10^4^ mM^-1^ min^-1^. The catalytic efficiency of I45A is reduced only 29-fold in comparison to wild-type, whereas the catalytic efficiency of I45Y is reduced 480-fold (Table 1). This is in contrast to monomeric TIMs from *P*. *falciparum*, *T*. *brucei* and *T*. *cruzi*, created by deletion of loop 3 that reduced their catalytic activity by 3 or 4 orders of magnitude [10, 11, 31].

**Table 1:** Catalytic parameters of ball and socket TvTIM mutants.

Description	K_m_ (mM)	K_cat_(min^—1^)	K_cat_/K_m_ (mM^-1^ min^-1^)	fold decrease in catalytic efficiency
TIM1WT	0.23 ± 0.02	7.98X10^4^	3.5X10^5^	1
I45G	1.78 ± 0.21	4.37 X10^3^	2.45 X10^3^	142
I45A	0.39 ± 0.04	4.7 X10^3^	1.19 X10^4^	29
I45V	0.30 ± 0.023	9.9 X10^4^	3.28 X10^5^	1.06
I45L	0.32 ± 0.021	1.14 X10^5^	3.52 X10^5^	0.99
I45F	0.521 ± 0.04	1.06 X10^3^	2.0 X10^3^	175
I45Y	1.29 ± 0.24	9.4 X10^2^	7.3 X10^2^	479.4
I45W	----	----	----	----

By incubating TvTIMs at different concentrations it can be predicted if activity is dependent or not on dimerization. The specific activity of the monomeric mutants is constant at a concentration between 5 to 100 ng per mL (S2 Fig), which implies that the observed catalytic activity is due to the activity of the monomer or that the dimer is not assembled at a concentration of 100 ng per mL

### Mutations in the ball display differential *in vivo* complementation

**Fig 3:**
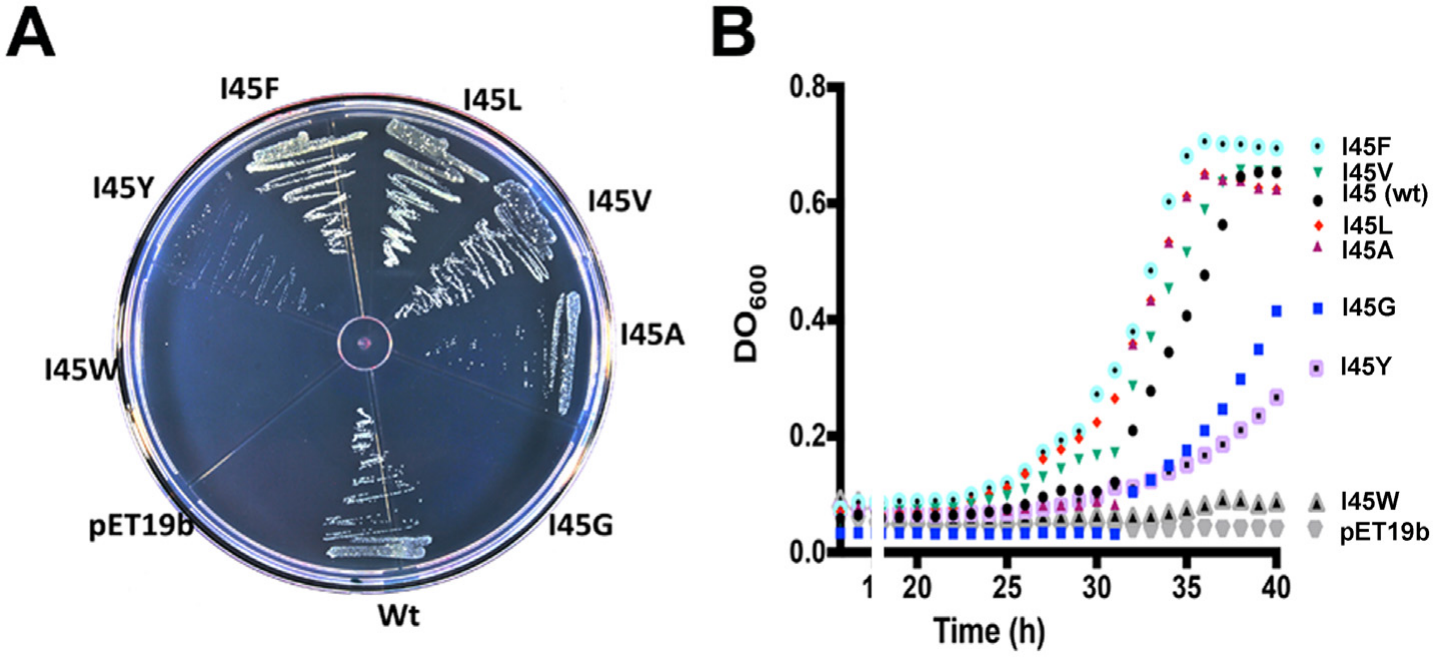
*In vivo* characterization of TIMs complemented strains on glycerol minimal media in the presence of IPTG. **(A)** Complementation of point mutants into an *E*. *coli* DE3 ΔTIM strain. Transformed *E*. *coli* were grown in plates with glycerol as a carbon source in M63 minimal media and 0.1mM of IPTG. *E*. *coli* transformed with plasmids containing wild-type TvTIM1 are able to complement. Mutants I45A, I45V, I45F and I45L complement with similar efficiency as wild type, whereas mutants I45Y contained less colonies, and no colonies appear after 48 hrs for I45G and I445W. **(B)** Growth kinetics of *E*. *coli* complemented strains in liquid minimal media. Growth rates of cultures grown in minimal medium. Bacteria transformed with plasmids containing wild-type TvTIM1 and mutants I45L, I45V, complement with similar efficiency, whereas, mutants I45A, I45F, I45Y and I45G present slower growth rates and no growth was observed with the tryptophan mutant and the empty vector.

Because of the decrease in catalytic efficiently observed in the monomeric mutants we were curious to assess the complementation of TvTIM mutants *in vivo*. An *E*. *coli* (DE3) ΔTIM strain [27] transformed with plasmids over expressing I45F, I45L, I45V, I45Y, and I45A mutants complemented in minimal media similarly than wild-type TvTIM1 after an incubation period of 48 h at 30°C. In contrast no colonies were present after a transformation with plasmids containing I45G and I45W mutants (Fig 3A). The growth rates of bacteria cultures in minimal medium indicates that bacteria transformed with plasmids containing wild-type and mutants I45L, I45V, I45A, I45F present similar growth rates, whereas I45Y and I45G present a slower growth rate. No growth was observed for I45W or an empty vector (Fig 3B).

### Monomeric TvTIMs unfold through a three state process

**Fig 4:**
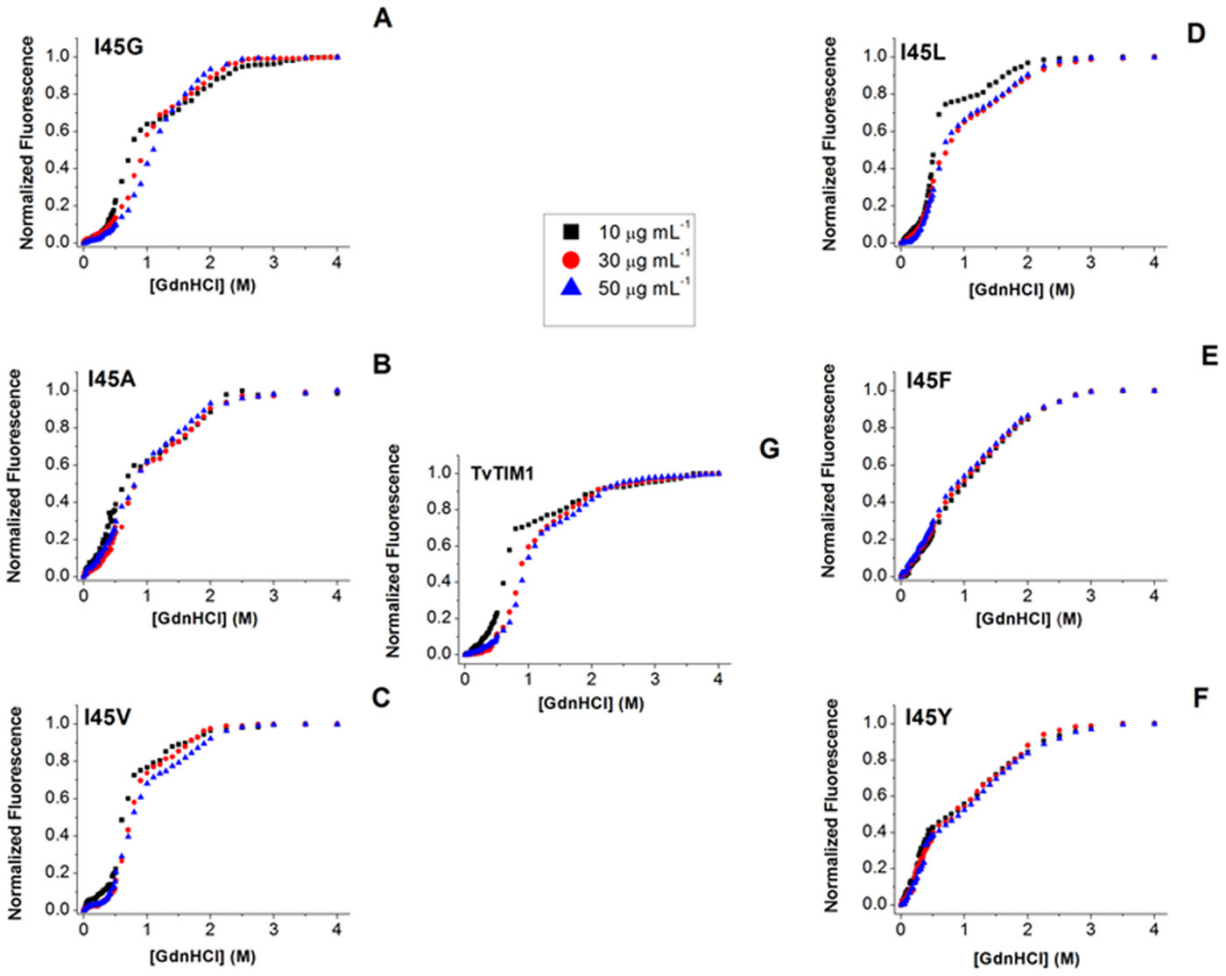
GdnHCl-induced denaturation profiles of TvTIM1 and mutants at residue 45, as measured by intrinsic fluorescence emission. (A) I45G, (B) I45A, (C) I45V, (D) I45L, (E) I45F, (F) I45Y and (G) Wild-type TvTIM1. Protein concentration was 10 (black squares), 30 (red circles) or 50 (blue triangles) μg mL^-1^ in 50 mM Tris-HCl pH 7.4 and 10 mM NaCl, at 25°C. Samples were excited at 280 nm and the data have been normalized for ease of comparison.

Unfolding studies indicate that TIMs monomers are barely stable [3, 32]. In contrast monomeric TvTIMs are populated as single species facilitating their chemical unfolding characterization. The GdnHCl-induced denaturation curves of monomeric mutants are observed in Fig 4 panels A, B, E and F.

Their transition profiles present a double sigmoidal curve. In all monomeric mutants with the exception of I45G, the pre-transition region is practically unappreciable. Considering that the monomeric species is the only populated species for I45F and I45Y and the most abundant for I45A and I45G, we used a three state denaturation model for monomeric proteins (*N* ⇔ *I* ⇔ *D*). Data were globally fit to Eq 8 (Material and Methods) Table 2.

**Table 2:** Thermodynamic Parameters for TvTIM1 and I45X mutants.

Monomeric Constructs Model: *N* ⇔ *I* ⇔ *D*				
	^a^ ^$\Delta G_{NI}^{H_{2}O}$^	^a^ ^,^ ^b^ ^$\Delta G_{ID}^{H_{2}O}$^	^a^ ^$\Delta G_{ND}^{H_{2}O}$^	
Construct	kJ mol^-1^	kJ mol^-1^	kJ mol^-1^	
I45G	17.8 (**±** 3)	52.8 (**±** 4)	70.6 (**±** 7)	
I45A	11.9 (**±** 3)	50.2 (**±** 5)	62.1 (**±** 8)	
I45F	11.3(**±** 2)	29.1 (**±** 3)	40.4 (**±** 5)	
I45Y	7.9 (**±**2)	26.2 (**±** 3)	34.1 (**±** 5)	
Dimeric Constructs Model: *N*_*2*_ ⇔ *2M* ⇔ *2I* ⇔ *2D*				
	^a^ ^$\Delta G_{N_{2}2M}^{H_{2}O}$^	^a^ ^$\Delta G_{MI}^{H_{2}O}$^	^a^ ^$\Delta G_{ID}^{H_{2}O}$^	^a^ ^,^ ^c^ ^$\Delta G_{N_{2}2D}^{H_{2}O}$^
Construct	kJ mol^-1^	kJ mol^-1^	kJ mol^-1^	kJ mol^-1^
TvTIM1	46.7 (**±** 3)	81.8 (**±** 5)	16.8 (**±** 1)	243.9 (**±** 9)
I45V	44.3 (**±** 2)	67.0 (**±** 4)	6.7 (**±** 1)	192 (**±** 7)
I45L	13.3 (**±** 3)	47.6 (**±** 2)	14.5 (**±** 3)	138 (**±** 8)

^a^ Measurements were made at 25°C and pH 7.4. Global analysis was performed with the non-linear, least-squares fitting program Origin, version 8.0. Standard deviations are indicated in parentheses.

^b^ calculated according to the relationship $\Delta G_{ID}^{H_{2}O}=\Delta G_{ND}^{H_{2}O}-\Delta G_{NI}^{H_{2}O}$

^c^ estimated using equation $\Delta G_{N_{2}2D}^{H_{2}O}=\Delta G_{N_{2}2M}^{H_{2}O}+2\Delta G_{MI}^{H_{2}O}+2\Delta G_{ID}^{H_{2}O}$

The free energy change of the first denaturation transition corresponding to the conformational transitions from the native state to the intermediate species, in the absence of denaturant for a standard state of 1 mol of monomer ($\Delta G_{NI}^{H_{2}O}$) indicates that I45G shows the highest value (17.8 kJ mol^-1^). Mutants I45A and I45F present similar $\Delta G_{NI}^{H_{2}O}$ values (11.9 and 11.3 kJ mol^-1^, respectively); in contrast I45Y shows the lowest value (7.9 kJ mol^-1^). The free energy change associated to the formation of the fully unfolded structure from the intermediate species ($\Delta G_{ID}^{H_{2}O}$) is similar for I45G and I45A variants (52.8 and 50.2 kJ mol^-1^, respectively). While I45F and I45Y show almost one half of this value (29.1 and 26.2 kJ mol^-1^, respectively). The free energy change corresponding to complete unfolding of the monomeric constructs ($\Delta G_{ND}^{H_{2}O}$), was calculated to be between 70.6 and 34.1 kJ mol^-1^. I45G seems to be the most stable monomeric TvTIM variant, followed by I45A, while I45F and I45Y are the most destabilized mutants. Remarkably, all monomeric mutants show free energy changes 3 to 10 times greater than values reported for human TIM [4].

### Dimeric TvTIMs fit to a four state model denaturation process

**Fig 5:**
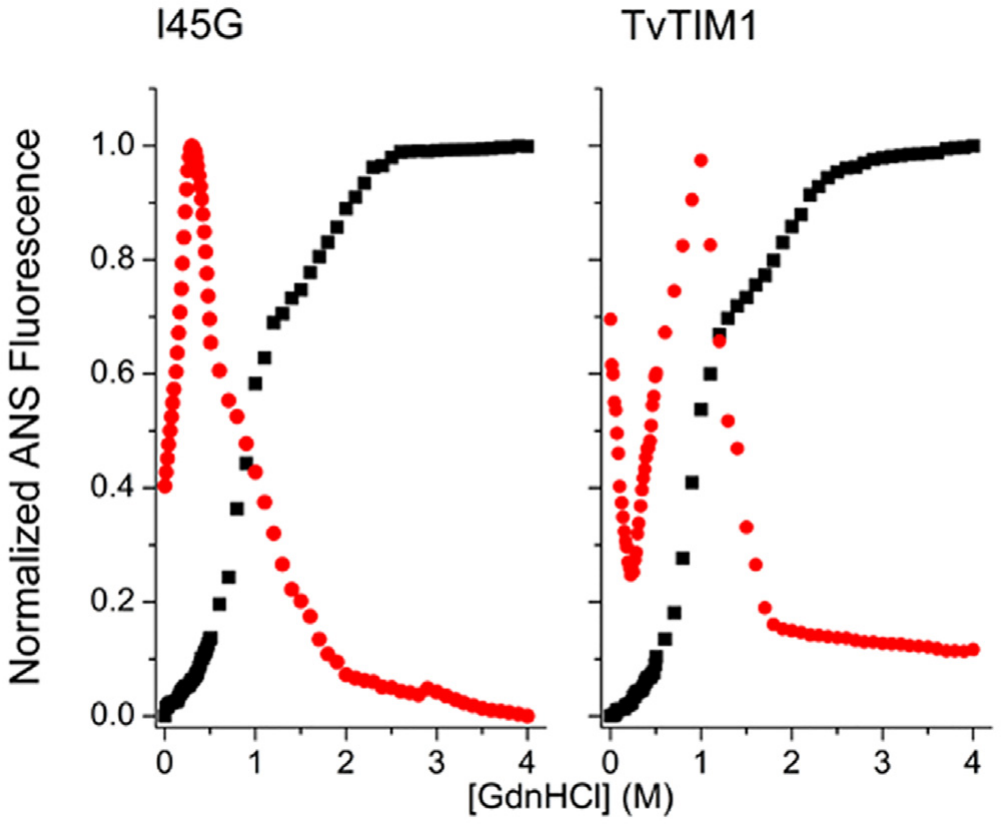
Unfolding profiles of TvTIM1 and I45G mutant monitored by Intrinsic (black squares) and ANS (red circles) fluorescence . The conditions are as described for Fig 4. The protein concentration was 50 μg ml^-1^. Data are normalized for ease of comparison.

The chemical denaturation transitions of wild-type and dimeric variants I45V and I45L are shown in Fig 4. All curves show double sigmoidal transitions and the datasets were fit to Eq 26 (Table 2). In contrast to monomeric constructs, the pre-transition region is doubtlessly detectable, and it was also detectable by changes in ANS fluorescence (Fig 5 and S3 Fig).

In this case, ANS binds more to native dimers, probably due to the partial exposure of hydrophobic regions nearby the monomer-monomer interface, than to folded monomers, that are unable to provide shielding from water. Partially unfolded monomers can be described as molten globules since they bind more ANS than native folded dimers and fully unfolded monomers. This kind of monomeric molten globules have been observed before in the equilibrium unfolding of triosephosphate isomerase from *Trypanosoma cruzi* induced by guanidinium hydrochloride (27).

Although I45V and I45L unfold by the same mechanisms than wild-type TvTIM1, there are subtle differences in their processes. I45V has similar free energy change of dissociation ($\Delta G_{N_{2}2M}^{H_{2}O}$ = 44.3 kJ mol^-1^) compared to wild-type ($\Delta G_{N_{2}2M}^{H_{2}O}$ = 46.7 kJ mol^-1^). Even though, important differences are observed in the free energy change of the two reaction-steps of the monomer unfolding, being 67.0 and 6.7 kJmol^-1^ for $\Delta G_{MI}^{H_{2}O}$ and $\Delta G_{ID}^{H_{2}O}$ respectively of I45V, compared to 81.8 and 16.8 for the wild-type protein. Meanwhile the less stable monomer correspond to the I45L, in this case important differences are observed in the free energy change of the dissociation step that is reduced up to 13.3 kJmol^-1^. The monomer of I45L is also destabilized compared to wild-type, in this case the estimated values of $\Delta G_{MI}^{H_{2}O}$ and $\Delta G_{ID}^{H_{2}O}$ were 47.6 and 14.5 kJmol^-1^ respectively.

### Crystal structures of mutant TvTIMs correlate with thermodynamic parameters

**Fig 6:**
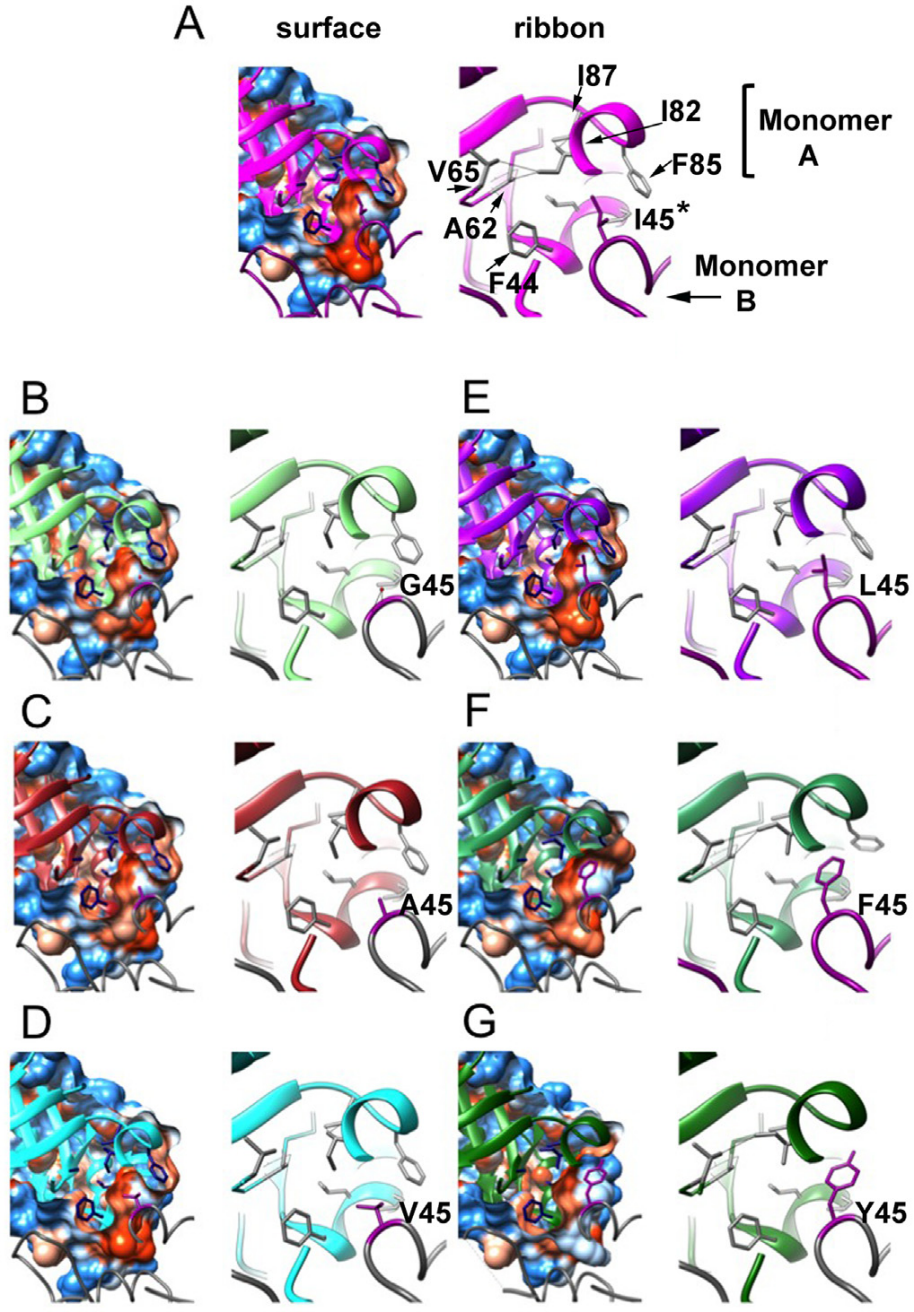
Crystal structures of TvTIM1 and point mutants at residue I45. Surface and ribbon representation of the ball and socket interaction at the dimer interface. Monomer A is represented as ribbons and monomer B is represented as coil. Residues of the socket (F44, A62, V65, I82, F85 and I87) of monomer A are shown in blue in the left panel and gray in the right panel, the ball residue (position 45) of monomer B is depicted in dark magenta in both panels. In the **left panel**, the cavity that is formed by residues of the socket is observed in a sliced view of the hydrophobicity surface of monomer A with the ball residue 45 of monomer B inside the cavity. Mutant I45G (B) shows a water molecule inside the cavity at H-bond distance from residues 45 and 46 of monomer B and residue 81 of monomer A. **Right panel**, a closer view of residues of the socket and the ball is shown. TvTIM1 structure (A) shows the most common rotamer for the side-chain of residue I82, whereas I45G (B), I45A (C), I45V (D) and I45L (E) mutants present the second frequent rotamer. I45F and I45Y enzymes display the third frequent rotamer for I82 residue.

Crystal structures of TvTIM1 mutants were solved by molecular replacement, all proteins crystallized in the P22_1_2_1_ space group with one molecule in the asymmetric unit that assembles as a dimer in the biologic unit of the crystal (S1 Table). The RMSD deviation in Cα-atom positions after superposition of mutants to the wild-type structure ranges from 0.13 to 0.24 Å with the exception of I45Y that shows a RMSD of 0.51 Å (Fig 6).

Mutant I45Y shows weak to none electron densities for residues 65 to 72. However there are some notable differences located at the dimer interface around position 45, where the Ile residue of the wild-type enzyme fits into a hydrophobic cavity resembling a "ball and socket". The side-chain of residue I82 shows three different rotamer conformations that correlate with the "size" of the residue at position 45. For the wild-type enzyme, with an isoleucine residue at position 45 (Fig 6 subunit A), the corresponding socket residue I82 (subunit B) shows its most common rotamer ("mt", with 60% frequency) [33]. In contrast, when I45 is mutated to glycine, alanine, valine, or leucine (Fig 6 subunit A), the corresponding socket residue I82 (subunit B) shows its second frequent rotamer (rotamer "mm", 15% frequency). One of the outcomes of this change is that the δ carbon atom of I82 side-chain moves towards the center of the "socket". This change at the socket, together with the repacking of the side-chains of residues F44, L47, A62 and F85 contribute to decrease the volume of the hydrophobic cavity as analyzed by the program POCASA [34] (Table 3).

**Table 3:** Cavities at the ball and socket interaction by mutations of residue 45.

Enzyme	Volume Å^3^
Ile45 TvTIM-1	46
I45G	22
I45A	21
I45V	42
I45L	30
I45F	30
I45Y	71
Val45-TvTIM-2 (3QST)	31

A closer view of the wild-type structure at the interactions of residue I82 with surrounding residues at the socket indicates distances in the range of 3.39 to 3.94 Å. Those distances were observed for either the δ carbon or the γcarbon-2 of I82 with the γ carbon-1 of V65, the β carbon of A62, the β carbon and γ carbon-2 of I87 and the γ carbon-1 of V77. With the change in the rotamer of I82 in the mutants I45G, I45A, I45V or I45L, the distance between the δ carbon of I82 and the β carbon of A62 change from 3.82 to 4.07–4.31 Å. A more dramatic change was identified for the interaction between the δ carbon of I82 and the δ carbon-1 of V65 that varies from 3.94 Å in the wild-type enzyme to 5.06–5.33 Å in TvTIM1 I45 mutants I45G, I45A, I45V or I45L.

The structure of I45G shows a water molecule (W420) placed in the hydrophobic cavity that is within a hydrogen bond distance of residues 45 and 46 of subunit A and residue 81 of subunit B. The distance of W420 to the nitrogen of residues 45 and the nitrogen of residue 46 of subunit A is 3.03 and 3.53 Å respectively, whereas the distance to the sulfur atom of M81 of subunit B is 3.26 Å. I45F and I45Y mutants present the third frequent rotamer for residue I82 (rotamer "pt", 13% frequency) in the "socket" and both mutants display longer distances between residue I82 and residues I87 and V77 that change from 3.39–3.94 Å to 5.04–5.93 Å. Furthermore, I45Y mutant shows a distance of 4.31 Å between I82 and A62.

**Fig 7:**
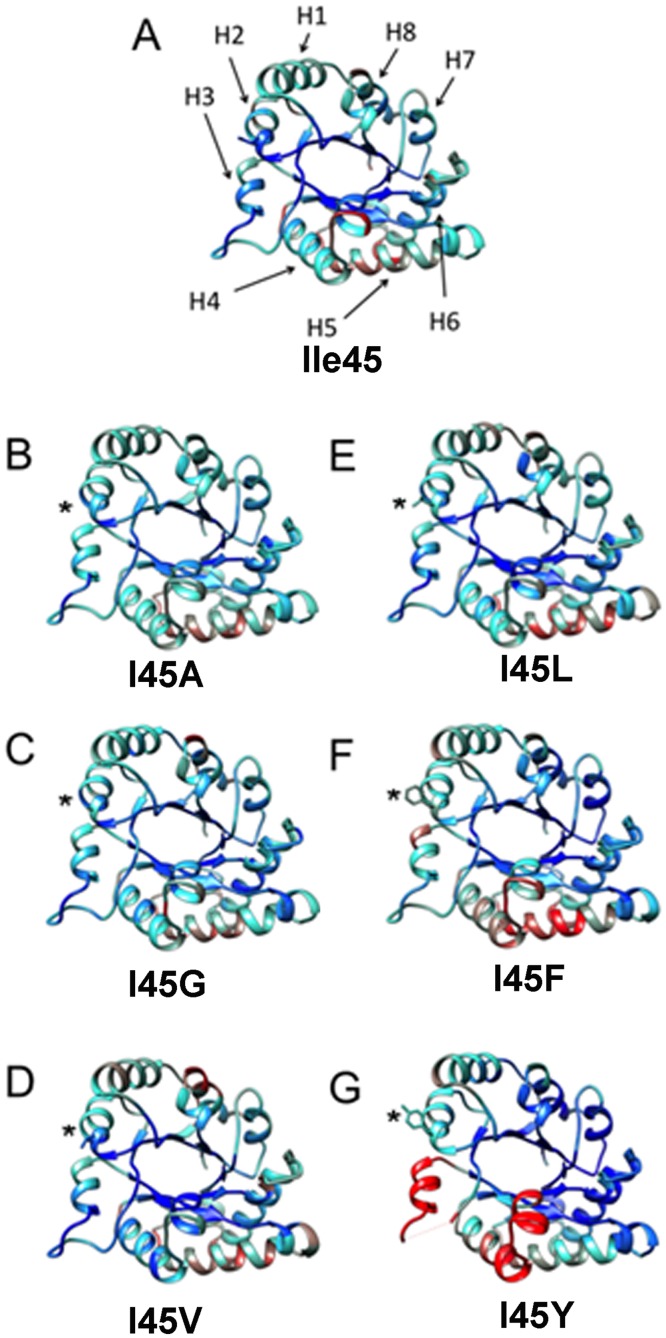
Comparison of the normalized B-factor values of TvTIM1 and mutants at residue 45. Crystal structure of the wild-type enzyme and the mutants ribbons indicating the variation of the normalized B-factors (A) TvTIM1 structure, the mean value of the normalized B-factor is represented in cyan, the lower value is blue, and the highest B-factor value is red. (B) I45G, (C) I45A, (D) I45V, (E) I45L, (F) I45F and (G) I45Y. The position of the eight α-helices of the (β-α)_8_ barrel fold is indicated by arrows (H1–H8) on TvTIM1 structure (A).

Another significant structural change at the residues in the hydrophobic cavity is present at residue F85. In the wild-type enzyme and TvTIM1 mutants with smaller side-chain residues at position 45, I85 delimits the exit of the hydrophobic cavity (Fig 6). As a result of the presence of a bulky residue at position 45 in mutant I45F, I85 side-chain is seen shifted away, by 4.61Å for the ξ carbon of F85 compared to the wild-type structure (Fig 6). A comparison of the normalized B-factors for TvTIM1 and residue 45 mutants indicates that this mutation did not increase flexibility/disorder in the neighbor residues (Fig 7).

However an increase in flexibility is observed in α-helices 3, 4 and 5 and in loop3 especially for I45F and I45Y mutants (Fig 7). Residues V77, M81, I82, and F85 of α-helix 3 are part of the socket in which residue 45 of the neighbor molecule attaches and loop 3 is responsible for a large surface area interaction between monomers. As the location of these structural elements is at the dimer interface, our data indicates that the character of the ball forces the socket to re-accommodate, and that the increase in thermal motion observed for monomeric mutants is a consequence of the new conformation of the socket. From the monomeric mutants, the I45Y present more dramatic differences. The crystal structure of I45Y lacks electron density for residues flanking F66 to F73 of the loop 3 (S4 Fig). Furthermore, V77 is part of loop 3 that interdigitates with the neighbor monomer. The fact that a mutation of a mutation of residue 45 generates high mobility of a region of loop 3 located at ~ 20 Å of distance is explained because of a rearrangement of residue V77 that reaccommodates to allow the packing of Y45 and thus distorted loop 3 or because the loop 3 is intrinsically disorder in a monomeric enzyme and cannot fit the neighbor molecule that presents higher B-factors in α-helices 4 and 5.

### Substrate binding induces dimer formation as measured by partial proteolysis and Analytical Ultracentrifugation

**Fig 8:**
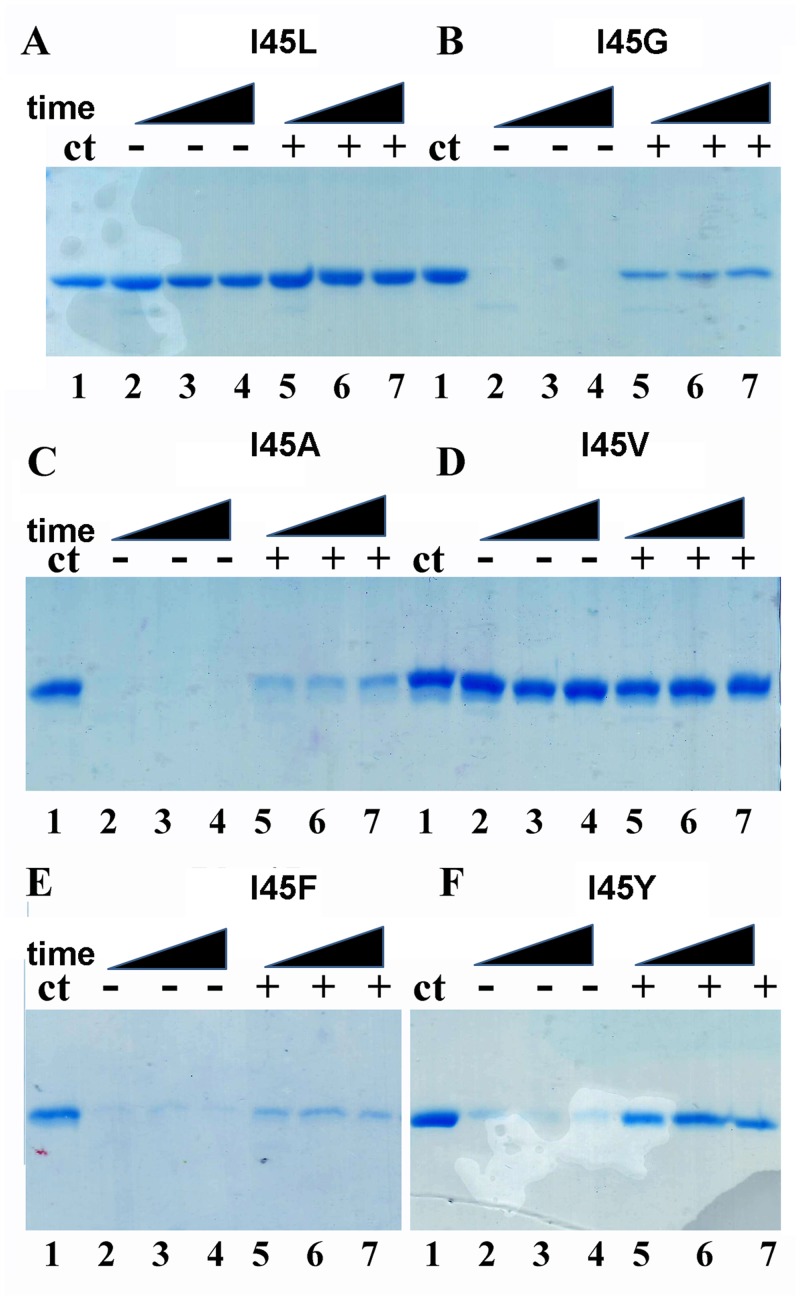
Monomeric TvTIMs are partially resistant to proteolysis upon 2PGA incubation. Time course experiment showing the partial proteolysis patterns of TvTIM mutants in the absence and in the presence of 2PGA. Dimeric TvTIMs are resistant to trypsinolysis with or without substrate analog (A and D). However monomeric TvTIMs are susceptible to trypinolysis in the absence of 2PGA but a small population of monomeric TvTIMs become resistant to after incubation with 2PGA (B,C,E,F). The extend of proteolysis is derived from the comparison to the control protein without added protease (lane 1).

As monomeric TvTIMs display high enzymatic activity and assemble as a dimer in the crystal enviroment, we wonder if the addition of a substrate analog would alter their oligomeric state in solution. A partial proteolysis experiment from 5 to 60 minutes was performed in the presence of 2 mM of the competitive inhibitor 2PGA (Fig 8).

Dimeric TvTIMs are resistant for proteolysis with or with the addition of 2PGA. In contrast monomeric enzymes are readily degraded during the time course of the reaction in the absence of 2PGA. In the presence of 2 mM 2PGA the digestion patter of dimeric enzymes is altered and a small population of the monomeric mutants presents resistance to proteolysis. This result may suggest that the addition of 2PGA alters the oligomeric state or produced a conformational change in the monomeric mutants that renders them resistant to proteolysis.

**Fig 9:**
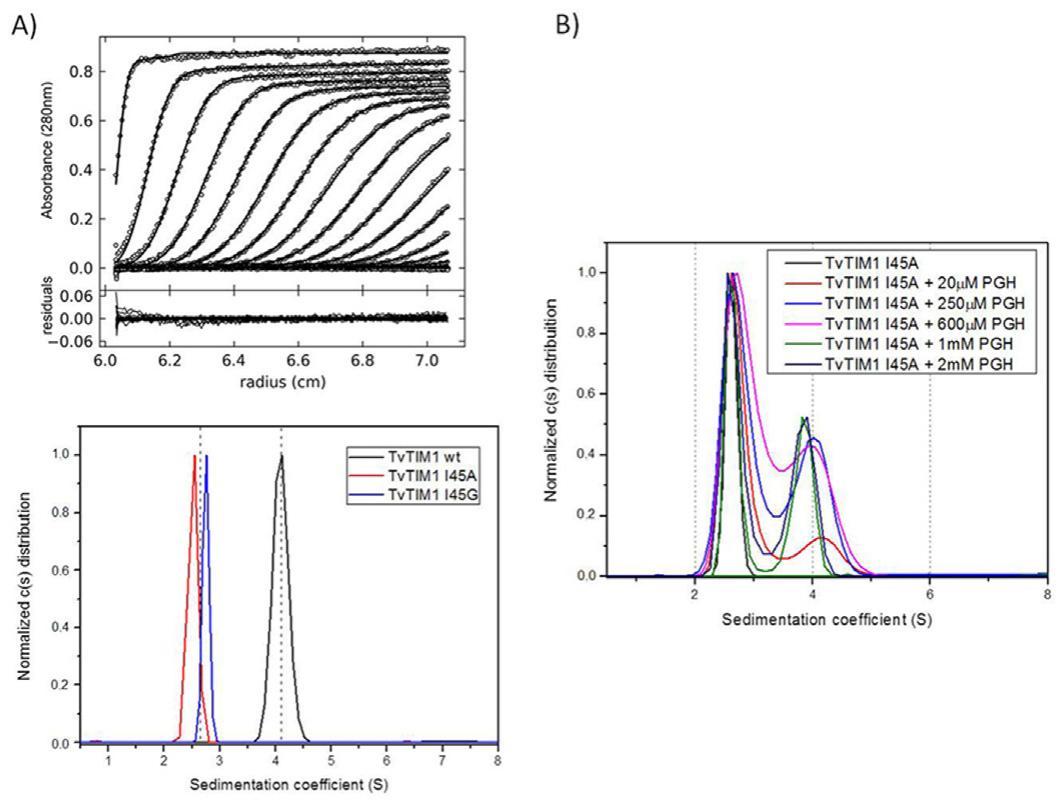
Substrate dependent dimerization of monomeric TvTIMs. (A) Trace of absorbance at 280 nm of TvTIM1 during Sedimentation Velocity experiment at the upper panel followed by the residuals bitmaps. Symbols correspond to experimental data and lines are the results fitted to the Lamm equation using Sedfit [28]. The lower panel shows continuous (c(s)) distribution of wild-type TvTIM1 (black curve) and monomeric mutants I45A (red) and I45G (blue). The left dashed line indicates monomer position whereas the right one indicates dimer. (B) Oligomeric states of I45A mutant in the presence of increasing concentration of PGH. Continuous (c(s)) distribution of I45A mutants in 20 mM Tris-HCl pH 8.0 plus 50 mM NaCl buffer. The distributions of protein without substrate are shown in black lines; the ones with 20 μM of PGH are shown in red lines, with 250 μM of PGH in blue lines, with 600 μM of PGH in pink lines, with 1000 μM of PGH in green lines and with 2000 μM of PGH in dark blue lines.

In order to understand if the proteolysis resistance of the monomeric mutants is due to the formation of a dimer, we performed sedimentation velocity experiments using wild-type TvTIM1 and I45A and I45G mutants over a concentration range from 7 to 35 μM show that the TvTIM1 is a dimer in solution, whereas I45A and I45G are monomers (Fig 9A).

In order to study if dimer assembly could be assisted by substrate binding, we employed increasing concentrations of the transition state analog phosphoglycolohydroxamate (PGH) from 20 to 2000 μM (Fig 9B). At a concentration of 20 μM of PGH 17% of the population of I45A is populated as a dimer and the appearance of the dimeric species reaches a maximum of 46% at a concentration of 2000 μM of PGH (Table 4).

**Table 4:** AUC data analysis for I45A mutant in 20 mM Tris-HCl.

	Sedimentation coefficient (S)	Molecular Mass (kDa)
**I45A**	2.6 ± 0.3	23 ± 2
**I45A + 20μM PGH**	2.7 ± 0.3	23 ± 3 (83%)
	4.1 ± 0.4	43 ± 6 (17%)
**I45A + 250μM PGH**	2.7 ± 0.2	23 ± 3 (62%)
	3.9 ± 0.3	41 ± 5 (38%)
**I45A + 600μM PGH**	2.7 ± 0.3	23 ± 3 (62%)
	4.0 ± 0.3	40 ± 5 (38%)
**I45A + 1000μM PGH**	2.6 ± 0.2	21.8 ± 0.7 (56%)
	3.8 ± 0.4	38.0 ± 0.9 (44%)
**I45A + 2000μM PGH**	2.5 ± 0.3	25 ± 4 (54%)
	3.7 ± 0.4	44 ± 6 (46%)

pH 8.0 and 50 mM NaCl with different concentrations of PGH

### A cross-linked monomeric mutant is as active as wild-type TvTIM1

**Fig 10:**
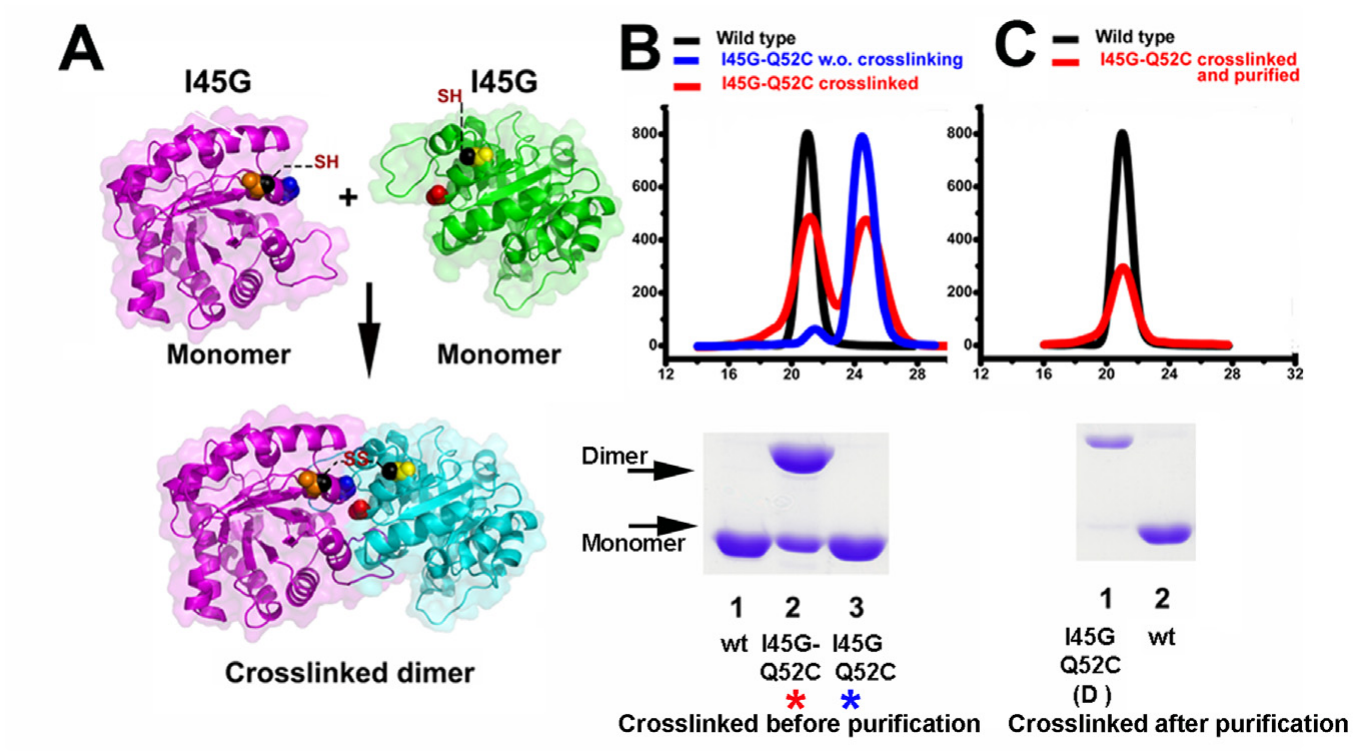
Disulfide Cross-linker induced dimerization of I45G mutant. **(A)** Localization of the engineered I45G-Q52C double mutant. The upper panel represents the monomeric I45G-Q52C double mutatn that may assemble as a dimer by effect of a cross-linking reaction (lower panel) **(B)** Gel filtration elution profiles and SDS-PAGE analysis for wild-type and I45G-Q52C double mutant before and after cross-linking. Approximately two thirds of the I45G-Q52C double mutant cross-linked in to a dimer as assessed by SDS-PAGE (lane 3 bottom panel). The differences between the percentage of assembled dimer by SDS-PAGE and gel filtration may due to a differential in the exposure of aromatic residues between the monomer and dimer **(C)** Gel filtration elution profiles and SDS-PAGE for wild-type and purified cross-linked I45G-Q52C double mutant. The gel filtration step efficiently separates the cross-linked I45G-Q52C double mutant from the unreacted protein (lane 1 bottom panel). The elution profiles have been normalized to ease comparison.

We reasoned that the reduced activity of the monomeric mutant is a consequence of deficiencies in dimer formation and that substrate binding of other forces may be able to restore the dimer. For instance, the crowding effect increases the formation of dimeric TIMs from from unfolded monomers [35]. Chemically cross-linking has been used to stabilize monomeric variants of Cu/Zn superoxide dismutase [36]. Mutant I45G reduces its catalytic efficiency 142-fold in comparison to wild-type TvTIM1 (Table 1) and we reason that a covalent cross-linking of the monomeric I45G mutant may increase its activity. For site-directed mutagenesis we selected residues Q52 and K53 since their C-β between monomers are 11 and 13 Å separated. We assayed dithio-bismaleimidoethane (DTME) and 1,4-bismaleimidyl-2,3-dihydroxybutane (BMDB) as cross-linkers with space arms of 13.3 and 10.2 Å respectively (Fig 10A).

A cross-linking reaction induced dimerization of approximately 70% of the double mutant I45G-Q52C (Fig 10B) and approximately 30% for the I45G-K53C mutant (data not shown) as assed by gel filtration chromatography. The dimeric I45G-Q52C can be purified from the un-reacted monomeric population by gel filtration (Fig 10C). In order to understand if the assembled dimer restores enzymatic activity, we measured its activity in comparison to wild-type, Q52C and the I45G-Q52C before the cross-linking (Table 5).

**Table 5:** Catalytic parameters of I45G-Q52C double mutant before and after cross-linking.

Description	K_m_ (mM)	K_cat_(min^—1^)	K_cat_/K_m_ (mM^-1^ min^-1^)	fold decrease in catalytic efficiency
Wild-type	0.23 ± 0.02	7.98 X10^4^	3.5X10^5^	—
I45G	1.78 ± 0.21	4.37 X10^3^	2.45 X10^3^	142
Q52C	0.27± 0.04	7.1 X10^4^	2.62 X10^5^	1.3
I45Gly-Q52C	1.66 ± 0.13	3.95 X10^3^	2.38 X10^3^	147
I45G-Q52C Cross-linked	0.45 ± 0.06	6.7 X10^4^	1.5 X10^5^	2.3

The single mutant Q52C reduced its catalytic efficiency by 77% in comparison to wild-type TvTIM1, indicating the Q52C mutation did not drastically alter the catalytic properties of the recombinant protein. The double mutant I45G-Q52C presents a decrease of 147 times in its catalytic efficiency with respect to wild-type, this fold reduction is activity is similar to the 142-fold decrease observed for I45G mutant. However, the catalytic efficiency of the purified cross-linked I45G-Q52C mutant is reduced 2.3-fold in comparison to wild-type TvTIM1 (Table 5). Thus the cross-linking is able to modify a 147-fold reduction to a 2.3-fold reduction in enzymatic activity. This change in catalytic efficiency was only observed for the cross-linked dimeric species and not for the monomeric species, indicating that the activity is due to the formation of a dimer.

## Discussion

Dimeric TvTIMs assemble by a ball and a socket interaction, in which residue 45 (ball) fits into a hydrophobic cavity (socket) of the other monomer [22]. This mechanism resembles the mechanisms for Cro and GST dimer formation mechanisms [37, 38]. Herein we describe how the character of the ball defines whether TvTIM1 adopts a monomeric or dimeric structure.

### Unfolding pathway of monomeric and dimeric isoforms of TvTIM

In a previous study the experimental evidence for the chemical denaturation of TvTIM1 and TvTIM2 indicated that a three-state model was sufficient to fit to the double sigmoidal denaturation curves [22]. The first step of those profiles was attributed to the dissociation process and the unfolding of the monomeric intermediate species (I) was proposed to correspond to the second sigmoidal detected in the profiles. In this study we collect experimental data from monomeric constructs. As monomeric variants show double sigmoidal denaturation profiles, the first transition cannot be attributed to a dissociation into monomers, because I45A, I45G, I45F and I45Y are mainly or solely monomers in the native state. The main difference in the shape of the chemical denaturation profiles between monomeric and dimeric TvTIM variants is the pre-transition step. Therefore we postulated that the dissociation process is almost transparent to secondary and tertiary structural probes used in our studies (intrinsic fluorescence and far-UV CD). In view of this, we used a four state model involving two monomeric intermediates to describe the unfolding reaction of TvTIM1and its dimeric variants. We postulate that the unfolding pathway of dimeric TvTIM is a sequential process: During the first step dimers (N_2_) dissociate into monomers (*M*). These monomers conserve their secondary and tertiary structure, this step being undetectable by intrinsic fluorescence and far-UV CD spectroscopies, but could be detected by an increment in ANS-fluorescence in the pre-transition region, only observable in dimeric mutants (S3 Fig). The second step involves an important change in the hydrophobic environment of aromatic residues as judged from the increase in the SCM value. This step could also be detected by ANS fluorescence but is almost unappreciable by far-UV CD (S3 Fig). The second monomeric intermediate (*I*) in the unfolding pathway of TvTIM1, exposes aromatic residues, contains hydrophobic regions that are able to harbor ANS and keeps mostly the native-like secondary structure. The last denaturation step involves further exposure of tryptophanyl residues as well as the loss of secondary structure content. We have reanalyzed the denaturation profiles of wild-type TvTIM1 and obtained revised thermodynamic parameters considering our new findings. Also, we calculated thermodynamic data for dimeric variants for the first time considering the four-state denaturation model (Table 2).

### Dimeric constructs of TvTIM

Substitutions of residue I45 for valine and leucine were the only dimeric mutants obtained in this study. Leucine has similar van der Waals radius than isoleucine, but with different configuration. While isoleuce is a β-branched amino acid, leucine has a γ-branched side-chain. Wild-type TvTIM1 and mutant I45L show almost identical catalytic efficiencies and secondary structure. Notably, I45L is the less stable dimeric mutant, particularly the free energy change associated to dimer dissociation reduced about three times compared to wild-type. This indicates that the ball and socket mechanism depends not only in the side-chain length, but also in side-chain branching of the ball to exquisitely fit into the socket. This reduction of the ball volume was not sufficient to alter the dimer stability of TvTIM1, but it decreased the stability of the monomer by 25% as judged from the free energy changes associated to monomer unfolding (Table 2).

### Monomeric constructs of TvTIM

TIM monomers obtained by the deletion of loop 3 are unstable and present poor catalytic activity [10, 11, 31]. The thermodynamic parameters for the dissociation and unfolding reactions of TvTIM1 indicate that dissociation contributes to only 18% of the total free energy change of unfolding, respectively. The substitutions at position 45 for glycine, alanine, tyrosine and phenyalanine produced monomeric proteins, even at a concentration of 260 μM. Glycine and alanine have smaller side chains than isoleucine, thus the hydrophobic ball is not able to fill the socket cavity, and in consequence some favorable hydrophobic interactions for the subunit-subunit association are removed. Hence, although a small population of dimer was detected by gel filtration, the equilibrium was displaced towards the monomer and at lower concentrations the dimer was not populated. On the opposite, the aromatic residues tyrosine and phenylalanine bear larger side chains than isoleucine. These substitutions correspond to balls that are larger than the available space in the socket cavity. This means than the ball and socket mechanism does not tolerate large changes on the volume of the ball to fit into the socket. Even more, it seems that larger volumes of the ball compared to isoleucine are more deleterious for dimer formation.

A thorough inspection of the crystallographic structures indicates that the overall scaffold of the monomers is not altered. Changes in the side chain of the ball produced a concerted series of movements of the side chains of the socket, particularly of I82, resulting either in a tighter cavity, or an overpacked socket giving room to aromatic residues at position 45. Notably the catalytic efficiency of the monomeric mutants was reduced from 29 times for I45A to 480 times to I45Y times compared to the catalytic efficiency of the wild-type enzyme, whereas catalytic efficiencies reported for other TIM monomers reduce their catalytic efficiencies three to four orders of magnitude. The GdnHCl induced denaturation of TvTIM1 monomeric mutants unfold via a three-state model, similarly to each subunit of the dimeric constructs. The most stable monomer corresponds to I45G. In this case its crystallographic structure revealed the presence of a water molecule into the hydrophobic cavity that might establish an intra-subunit hydrogen bond network between residues 45 and 46. These interactions could be sufficient to increase the conformational stability of I45G mutant compared to the other monomers. Our data indicates that in contrast to archetypical TIMs, TvTIMs are stable and properly fold as monomers. In one hand the concentration kinetics indicate that ball and socket mutants are active as monomers, however it is intriguing that even at a concentration of 260 μM the monomeric mutants are not able to dimerize. Structural data indicates the assembly of TvTIM monomeric mutants as dimers, however the socket suffer rearrangements during the crystallization process.

### Substrate induced and cross-linking dimerization

Monomeric mutants may be populated as a mixture of monomers and dimers. For instance approximately 13% of the I45A mutant is a dimer at 260 μM as measured by gel filtration, but the dimeric species is not populated at a concentration of 26 μM as assessed by gel filtration and AUC. I45A is only 29-fold less active than wild-type TvTIM and is as effective as the wild-type enzyme in complementing an *E*. *coli* ΔTIM strain. The biological unit for all monomeric mutants is a dimer, as the crystallographic data was collected from crystals in the same space group than wild-type. In light of those observations we reason that monomeric mutants could be populated as dimers if they are guided by crystal packing or other forces. It is knows that substrate binding is able to induce protein dimerization [39–42]. Thus, we hypothesized that the high enzymatic activity of monomeric TIMs may be related to assembly-disassembly of the catalytic competent dimeric species. We decided to measure if the presence of the substrate analog PGH would increase the formation of the dimeric species by AUC. We use a concentration of 1 mg/ml (~38 μM) for these studies and found that the population of the dimeric species increases as the concentration of PGH increases. At 200 μM of PGH ~50% of I45A is populated as a dimer. The notion that substrate induces I45A dimerization suggests that a chemical cross-link between monomeric TvTIMs would favor the assembly of monomers into a catalytic competent dimer. An I45G-Q52C mutant alters its catalytic activity from a 147-fold decrease in catalytic efficiency without cross-linking to a 2.3-fold decrease in a cross-linked dimer. It is know that TIMs exists in a open-closed conformational equilibrium in which loop 6 reorients into the active site, and that PGH and other substrate analogs favors the transition from an open to a closed conformation [43–45]. Our data indicates that in TvTIMs the formation of stable monomers is indispensable for dimerization and indicates that induced dimerization accounts for the high enzymatic activity of monomeric TvTIMs. The exact mechanism by which this conformational change may alter the subunit interface to allow dimerization requires further structural studies. The serendipitous fact that monomeric TvTIMs are highly stable allows the discovery that TIM is an enzyme in which substrate induced its dimerization.

## Supporting Information

S1 Fig Logo sequence of triosephosphate isomerases from Archaea, Eukarya and Bacteria. The logo indicates the relative the relative frequencies of every residue at every position.
(TIF)

S2 Fig Catalytic activity of TvTIM in function to enzyme concentration. The specific activity (Units/mg) was measured at 5, 25, 50, 75 and 100 ng of enzyme for each monomeric mutants. The linear specific activity indicates that the observed enzymatic activity is not dependent of enzyme concentration.
(TIF)

S3 Fig Unfolding profiles of I45A, I45V, I45L, I45F, and I45Y mutants monitored by Intrinsic (black squares) and ANS (red circles) fluorescence. The conditions are as described for Fig 4. The protein concentration was 50 μg ml-1. Data are normalized for ease of comparison.
(TIF)

S4 Fig Ile45Tyr mutant produces a disorder loop3. Crystal structure of dimeric I45Y mutant. Residues Y45 are colored in red and magenta in each subunit. α 3 and 4 present increased B-factors and are colored in yellow and orange. The loop 3 of each subunit shows a break in electron density from residues F66 to F73.
(TIF)

S1 Table Data collection and refinement statistics. (DOCX)
