# Supplementary figures and images for: Substrate-Induced Dimerization of Engineered Monomeric Variants of Triosephosphate Isomerase from Trichomonas vaginalis
Source: PLoS One. 2015 Nov 30;10(11):e0141747. doi: 10.1371/journal.pone.0141747 (PMC4664265; doi:10.1371/journal.pone.0141747)

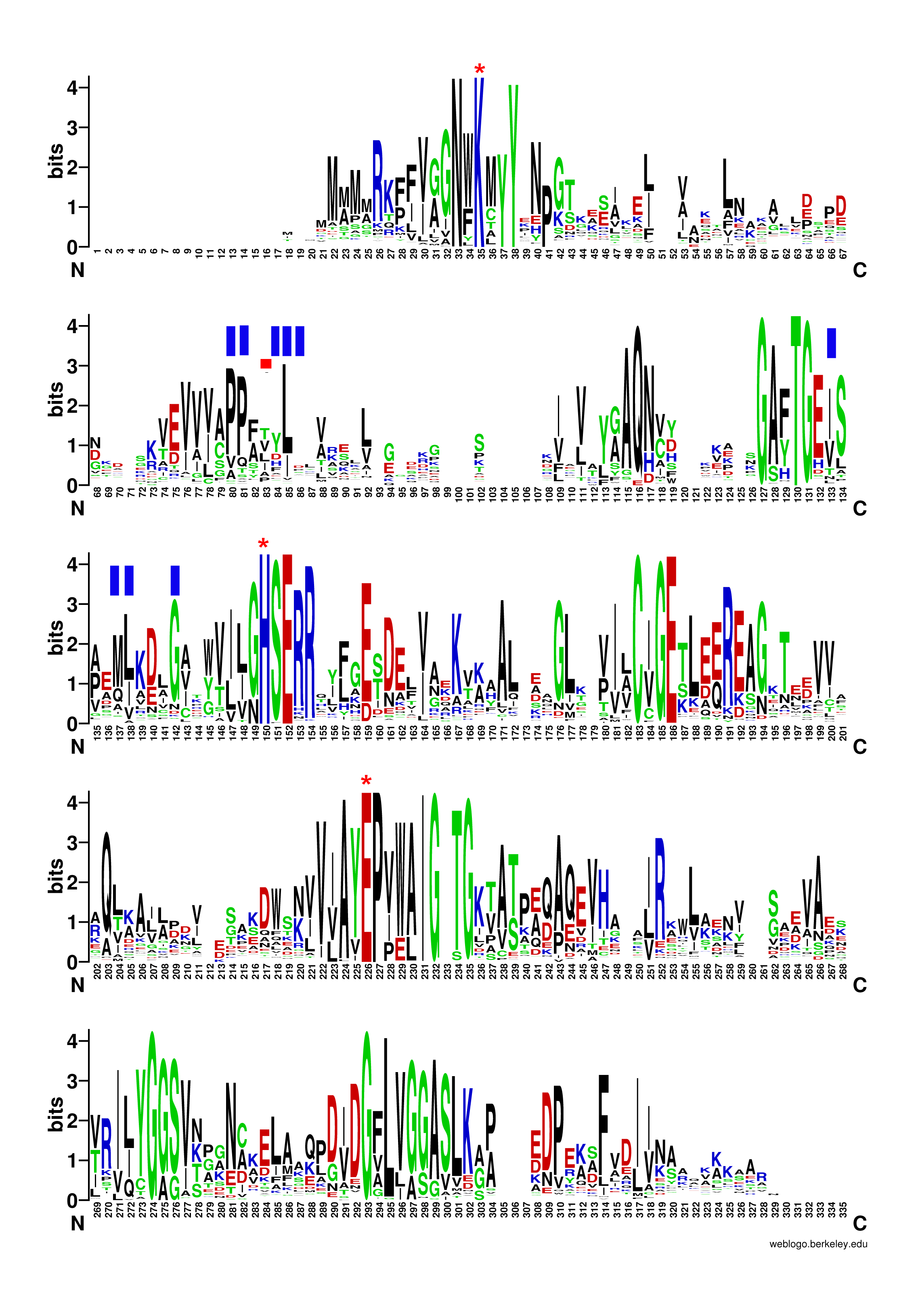

Supplement: S1 Fig — The logo indicates the relative the relative frequencies of every residue at every position. (TIF) [file pone.0141747.s001.tif]

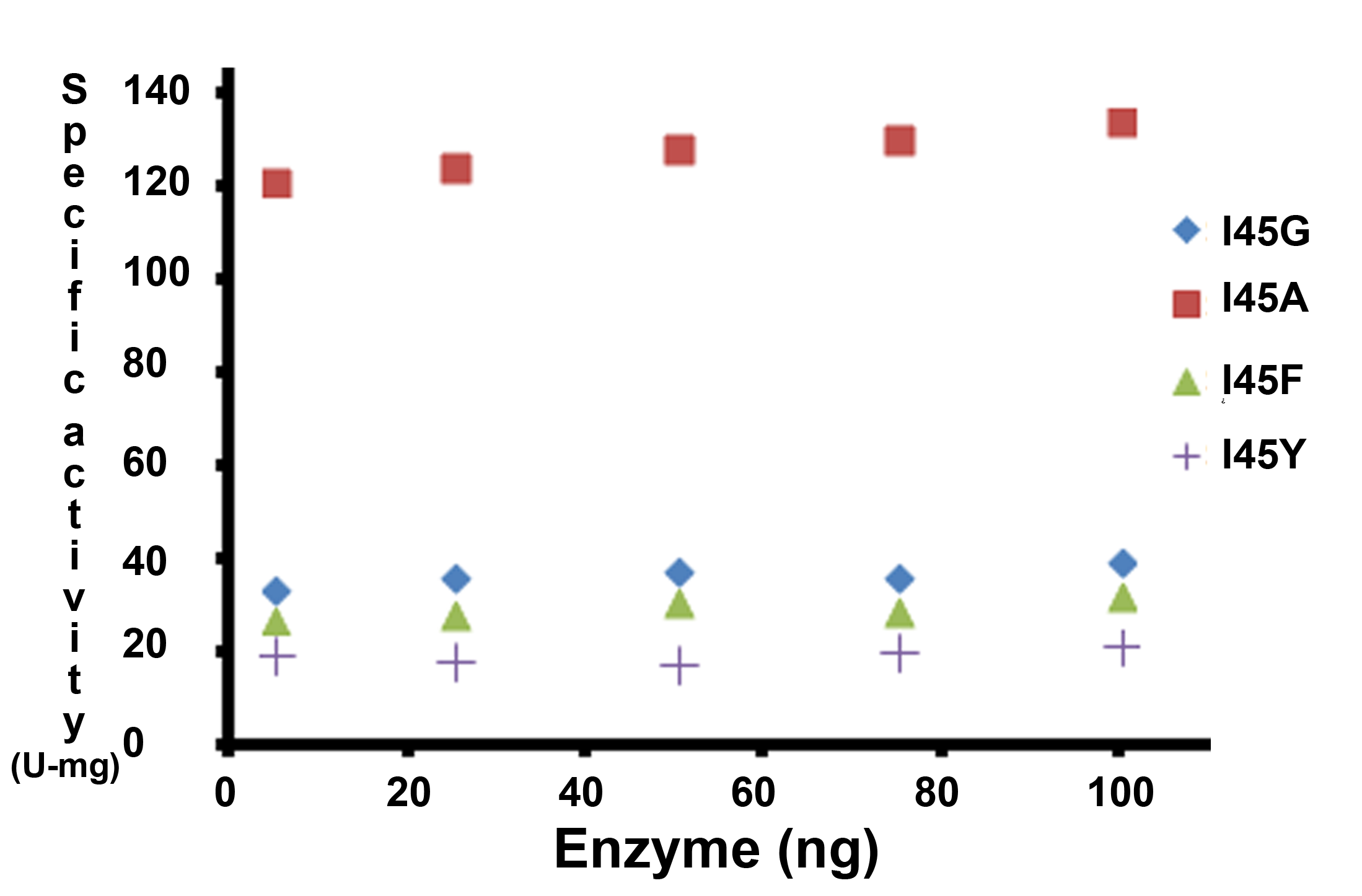

Supplement: S2 Fig — The specific activity (Units/mg) was measured at 5, 25, 50, 75 and 100 ng of enzyme for each monomeric mutants. The linear specific activity indicates that the observed enzymatic activity is not dependent of enzyme concentration. (TIF) [file pone.0141747.s002.tif]

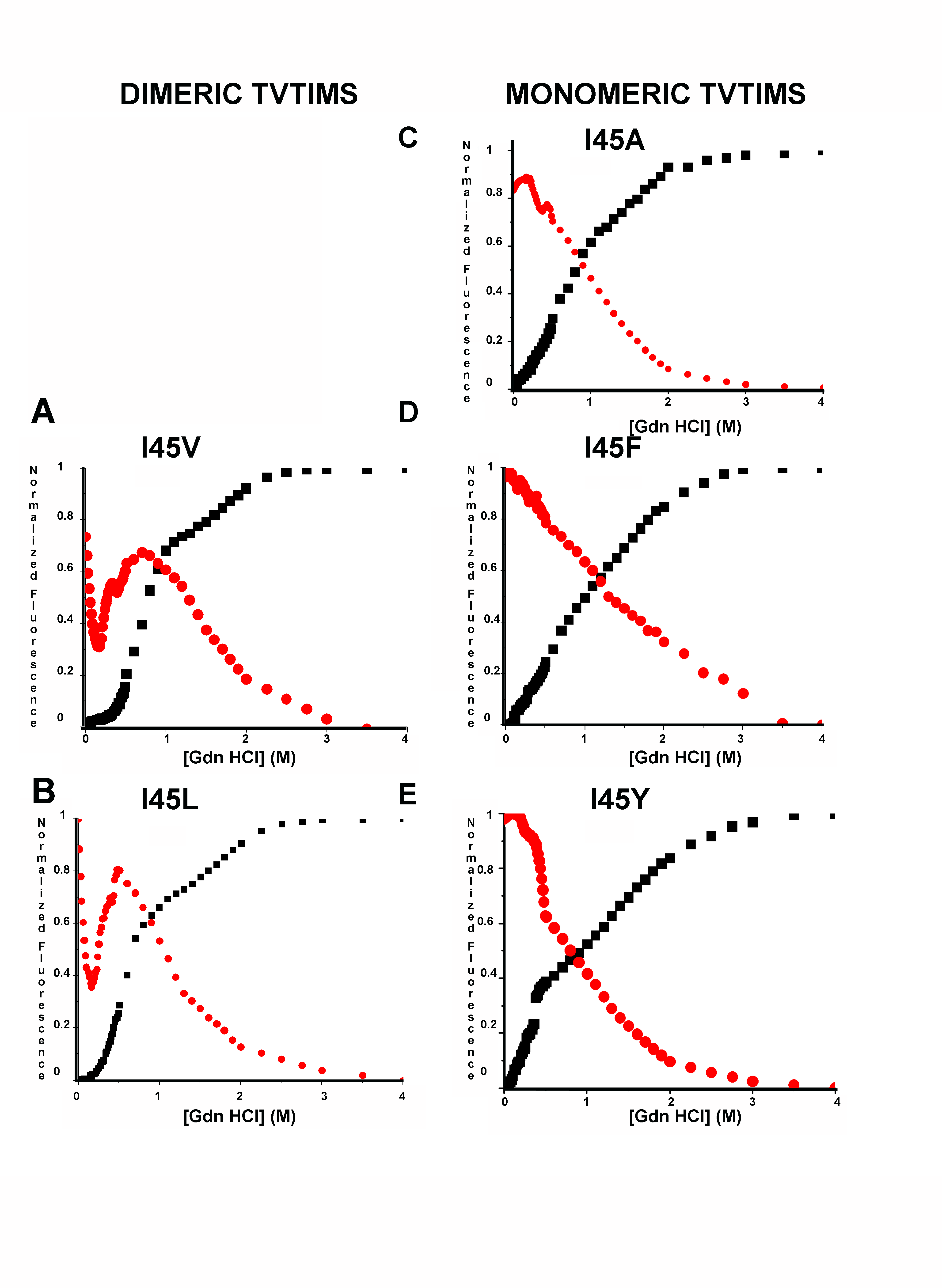

Supplement: S3 Fig — The conditions are as described for Fig 4. The protein concentration was 50 μg ml-1. Data are normalized for ease of comparison. (TIF) [file pone.0141747.s003.tif]

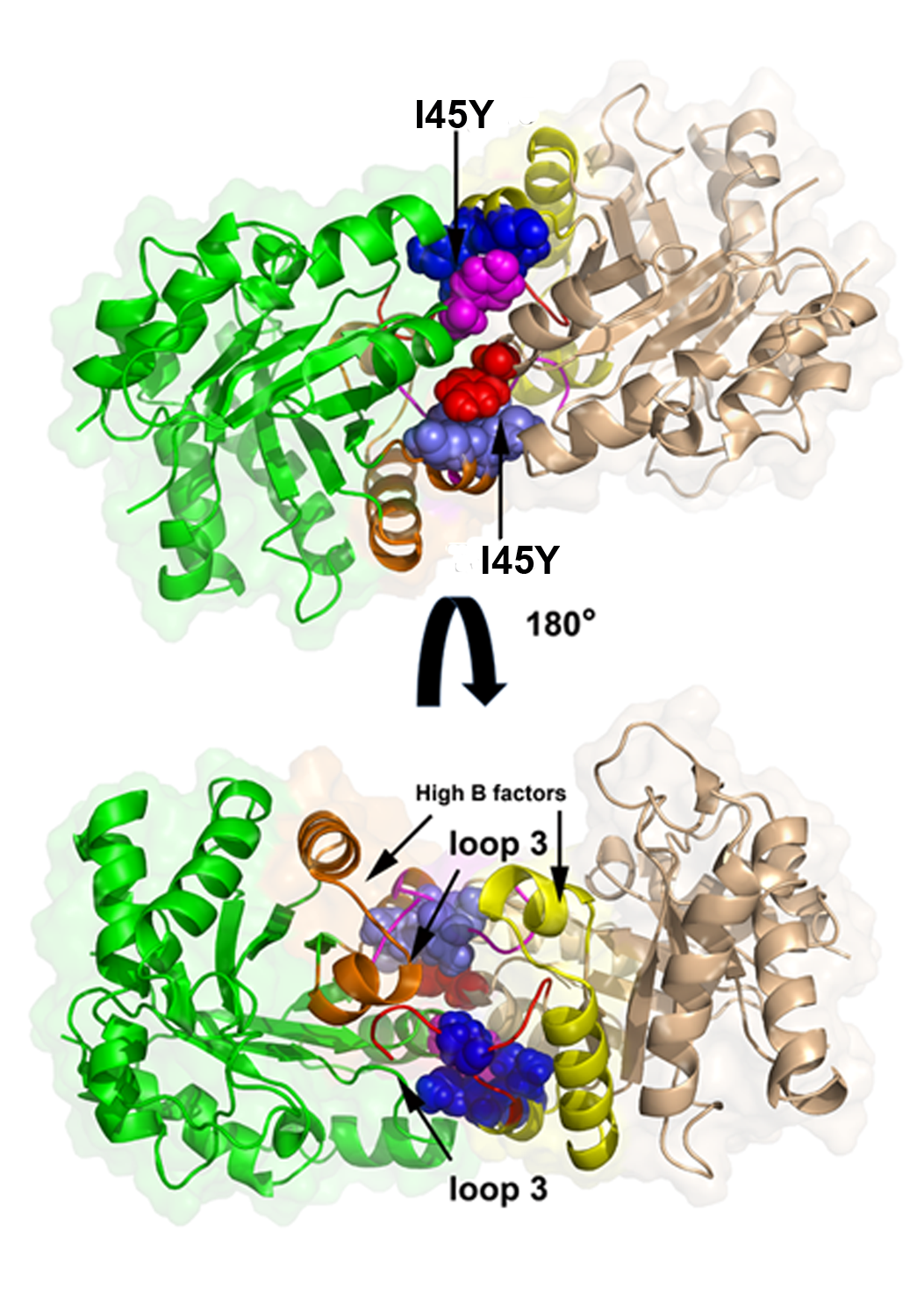

Supplement: S4 Fig — Crystal structure of dimeric I45Y mutant. Residues Y45 are colored in red and magenta in each subunit. α 3 and 4 present increased B-factors and are colored in yellow and orange. The loop 3 of each subunit shows a break in electron density from residues F66 to F73. (TIF) [file pone.0141747.s004.tif]
