# Supplementary material for: Substrate-Induced Dimerization of Engineered Monomeric Variants of Triosephosphate Isomerase from Trichomonas vaginalis
Source: PLoS One. 2015 Nov 30;10(11):e0141747. doi: 10.1371/journal.pone.0141747 (PMC4664265; doi:10.1371/journal.pone.0141747)
Supplement: S1 Table — (DOCX) [file pone.0141747.s005.docx]

| **Table S1. Data collection and refinement statistics** | | |  |  |  |  |  |
| --- | --- | --- | --- | --- | --- | --- | --- |
|  |  |  |  |  |  |  |  |
|  | I45G | I45A | I45V | I5 | I45L | I45F | I45Y |
| PDB code | 4O4W | 4O50 | 4O52 | 4O4V | 4O53 | 4O54 | 4O57 |
| *Data collection* |  |  |  |  |  |  |  |
| Wavelength (Å) | 1.542 | 1.542 | 1.542 | 0.98 | 1.542 | 1.542 | 0.98 |
| Space group | *P*22_1_2_1_ | | | | | | |
| Unit-cell parameters |  |  |  |  |  |  |  |
| a,b,c (Å) | 46.8, 56.0, 104.8 | 46.9, 56.8, 106.8 | 46.8, 55.9, 104.9 | 47.2, 56.8, 107.9 | 46.9, 56.0, 105.2 | 46.8, 56.2, 105.9 | 47.3, 56.4, 106.3 |
| Resolution (Å) | 56.0-2.35 (2.48-2.35)^a^ | 56.8-1.95 (2.06-1.95) | 55.9-1.95 (2.06-1.95) | 23.1-1.23 (1.30-1.23) | 56.0-2.0 (2.11-2.0) | 46.9-1.9 (2.0-1.9) | 49.8-1.78 (1.88-1.78) |
| No. of observations | 67,912 | 154,433 | 149,097 | 477,106 | 99,855 | 211,668 | 210,624 |
| No. of unique reflections | 11,987 | 21,518 | 20,768 | 84,500 | 19,121 | 21,301 | 27,186 |
| Mean I/σI | 11.7 (4.7) | 17.6 (5.7) | 15.7 (5.3) | 11.5 (4.8) | 18.8 (8.6) | 30.8 (5.6) | 19.1 (4.4) |
| Completeness (%) | 99.6 (98.6) | 100 (100) | 100 (100) | 99.5 (100) | 98.9 (96.6) | 94.6 (89.9) | 97.2 (81.7) |
| Redundancy | 5.7 (5.4) | 7.2 (7.2) | 7.2 (7.0) | 5.6 (5.6) | 5.2 (4.7) | 9.9 (5.3) | 7.7 (6.6) |
| *R*_merge_ (%) | 10.6 (34) | 8.4 (35) | 9.8 (37) | 8.6 (49) | 5.7 (15) | 5.2 (27) | 5.8 (33) |
| *Refinement* |  |  |  |  |  |  |  |
| Resolution (Å) | 52.4-2.35 (2.59-2.35) | 53.4-1.95 (2.03-1.95) | 49.3-1.95 (2.05-1.95) | 23.1-1.23 (1.26-1.23) | 49.4-2.0 (2.09-2.0) | 46.8-1.9 (1.96-1.90) | 35.3-1.8 (1.86-1.79) |
| No. of reflections |  |  |  |  |  |  |  |
| Work/Free set | 11,354 / 598 | 20,186 / 1,289 | 19,689 / 1,035 | 82,422 / 2,000 | 17,952 / 1,147 | 19,796 / 1,491 | 25,783 / 1,357 |
| *R*_work_/*R*_free_ (%) | 19.4 / 23.2 | 15.4 / 20.5 | 16.7 / 20.9 | 14.5 / 15.6 | 16.5 / 22 | 16.5 / 21.5 | 17.7 / 20.6 |
| Asymmetric unit content | Monomer | Monomer | Monomer | Monomer | Monomer | Monomer | Monomer |
| Averange B-factor (Å^2^) |  |  |  |  |  |  |  |
| Protein | 21.3 | 16.6 | 13.1 | 11.3 | 16.7 | 20.9 | 32.1 |
| Waters | 20.2 | 23.5 | 20.1 | 23.8 | 23.3 | 27.7 | 34 |
| RMSD from ideality |  |  |  |  |  |  |  |
| Bond lengths (Å) | 0.011 | 0.017 | 0.017 | 0.015 | 0.014 | 0.015 | 0.015 |
| Angles (deg.) | 1.17 | 1.4 | 1.44 | 1.48 | 1.33 | 1.4 | 1.44 |
| Ramachandran statistics | |  |  |  |  |  |  |
| Favored (%) | 97 | 98 | 97 | 98 | 97 | 97 | 99 |
| Outliers (%) | 0.4 | 0 | 0 | 0 | 0 | 0 | 0 |
| ^a^ Values in parentheses are for highest-resolution shell | | | | | | | |
| *R*_free_ was calculated from a set of randomly selected reflections (2-6%) and were not included in the calculation of the *R*_work_ values | | | | | | | |
